# A Reproducible and Explainable Deep Learning Framework for Dermoscopic Image Analysis in Skin Cancer

**DOI:** 10.3390/jcm15197422

**Published:** 2026-09-24

**Authors:** Abdulvahap Pınar, Fatma Hilal Yağın, Cemil Çolak, Burak Yağın, Sarah A. Alzakari, Amal K. Alkhalifa, Abedelmalek Kalefh Tabnjh

**Affiliations:** 1 Rectorate Unit, Adıyaman University, Adıyaman 02040, Türkiye; apinar@adiyaman.edu.tr; 2 Department of Biostatistics, Faculty of Medicine, Malatya Turgut Ozal University, Malatya 44210, Türkiye; 3 Biostatistics and Medical Informatics, Faculty of Medicine, Inonu University, Malatya 44280, Türkiye; cemil.colak@inonu.edu.tr (C.Ç.); burakyagin14@gmail.com (B.Y.); 4 Department of Computer Sciences, College of Computer and Information Sciences, Princess Nourah Bint Abdulrahman University, P.O. Box 84428, Riyadh 11671, Saudi Arabia; saalzakari@pnu.edu.sa (S.A.A.); akalkalifh@pnu.edu.sa (A.K.A.); 5 Department of Cariology, Institute of Odontology, The Sahlgrenska Academy, University of Gothenburg, 413 90 Gothenburg, Sweden; 6 Department of Applied Dental Sciences, Faculty of Applied Medical Sciences, Jordan University of Science and Technology, Irbid 22110, Jordan; 7 Department of Pathology, Saveetha Medical College and Hospital, Saveetha Institute of Medical and Technical Sciences, Chennai 602 105, Tamil Nadu, India

**Keywords:** melanoma, dermoscopy, deep learning, explainable AI, external validation, model selection bias, calibration

## Abstract

**Background/Objectives:** Cutaneous melanoma caused over 331,000 new cases and 58,000 deaths worldwide in 2022, with five-year survival falling from 99.4% in localised disease to 35.6% after distant metastasis. Most dermoscopic deep learning studies report headline accuracy without addressing data leakage, calibration, or how their comparisons are constructed. This study asks how much of that performance survives a protocol designed to remove the optimism. **Materials and Methods:** Six classification architectures were trained on HAM10000/ISIC 2018 (10,015 images, seven classes) at a fixed resolution and with fixed ImageNet-1k pretraining, the architecture confounding with neither. Five segmentation networks were trained alongside MedSAM and evaluated separately under reference-derived and fully automatic box prompts. Lesion-level StratifiedGroupKFold prevented duplicate-lesion leakage; temperature scaling used an inner-validation partition, never the test fold. Models were compared across outer folds (N = 5), saliency localisation against a fixed centred-disc baseline, and generalisation on BCN20000 (15,172 images). **Results:** The six configurations were statistically indistinguishable (Friedman χ^2^ = 7.057, *p* = 0.216; balanced accuracy 0.664–0.700), so no best architecture could be identified. MedSAM ranked first with reference-derived prompts (Dice 0.9547) and last with automatic prompts (0.7729), 16.7 points below a plain U-Net, its advantage reflecting prompt provenance rather than the model. All three class activation methods scored below the fixed disc on mask-IoU (0.2531–0.3512 against 0.4745). Externally, balanced accuracy fell 25 points and the ranking reversed, while AUC held at 0.80–0.84; recalibration helped only two of six models. **Conclusions:** Under a protocol that isolates each compared factor, the differences that such studies report do not survive. Cross-cohort loss is large and not reliably repaired by recalibration; the leverage lies in evaluation methodology, not architectural novelty.

## 1. Introduction

Cutaneous melanoma is the seventeenth most prevalent cancer in the world and the worst kind of skin cancer. According to the most current GLOBOCAN forecasts, 331,722 new cases and 58,667 melanoma-related deaths were observed globally in 2022, with the highest age-standardised incidence rates grouped in high human development index nations [1]. The American Cancer Society anticipated 212,200 new melanoma cases and 8430 disease-related deaths in the United States by 2025, representing a 42% rise in invasive cases since 2015 [2]. Five-year relative survival surpasses 99% for localised illness but drops to 35.6% when distant metastases develop [2,3], a sixty-percentage-point disparity that necessitates early, precise identification at the heart of any reasonable public health policy.

However, access to qualified dermatologists remains inconsistent. Vaidya and colleagues report that a substantial share of the United States population aged 65 and over lives in counties without a Medicare-participating dermatologist [4]; we cite this figure as the reference reports it rather than generalising it to the whole population. In this context, AI-augmented diagnostic tools have repeatedly demonstrated meaningful clinical value: two recent meta-analyses report that AI augmentation increases clinician sensitivity from 74.8% to 81.1% and specificity from 81.5% to 86.1%, with non-dermatologists and primary-care providers showing the greatest gains [5]. DermaSensor, the first standalone AI enabled skin cancer detection device for primary care, gained de novo approval from the US Food and Drug Administration on 17 January 2024 and reported a sensitivity of 95.5–96% with a negative predictive value of 96.6% at a specificity of 20.7% in its pivotal primary-care study [6,7]. As a result, the translational environment has shifted from theoretical to practical, necessitating more methodological rigour in academic algorithm development investigations.

It is also worth noting that dermoscopic presentation is not uniform within melanoma. Genetically defined subgroups differ in their clinical and dermoscopic phenotype; carriers of the MITF p.E318K variant, for instance, show distinguishable dermoscopic patterns in both melanomas and dysplastic naevi [8]. A model trained against a single label “melanoma” is therefore not learning a homogeneous target, a point we return to in Section 4.4.

Despite substantial advances in convolutional and transformer-based topologies, various persistent issues continue to undermine the clinical credibility of computer-aided dermoscopy systems. First, image quality remains a widely cited factor affecting downstream performance; hair artifact removal, contrast-limited adaptive histogram equalisation (CLAHE) and Shades-of-Gray colour-constancy transformation are all reported to improve accuracy or sensitivity on classical benchmarks [9,10,11,12]. We note that these are reports from the literature and that the present study does not itself measure the contribution of individual preprocessing modules (Section 4.4). Second, lesion-level data leakage in HAM10000—the canonical benchmark—has been documented in detail: the dataset contains only 7470 unique lesions across its 10,015 images, and image-level random splits produce an apparent accuracy bias of 1–3 percentage points higher than proper lesion-level group splits [13,14]. Third, calibration is nearly always overlooked: most published systems are consistently overconfident, with expected calibration errors (ECEs) of 0.10–0.13 [15], which is inconsistent with regulatory requirements for artificial intelligence (AI) as a medical device. Fourth, statistical comparison is usually restricted to point-estimate “leaderboard” reporting without bootstrap confidence intervals, paired tests (McNemar, DeLong), or proper multi-classifier techniques (Friedman with Nemenyi post hoc) [16,17]. Fifth, explainable AI is often given qualitatively without regard for fidelity, sensitivity, or sanity-checking [18,19]. To these five we add a sixth, which motivates the present study: the comparisons themselves are rarely constructed so that the reported difference can be attributed to the factor named. Architectures are compared while input resolution, pretraining corpus and parameter count vary together; prompted and unprompted segmentation models are placed in the same table; a best model is selected based on the evaluation that is then reported as its performance; and correlated repetitions are counted as independent observations. Each of these inflates apparent differences in the same direction.

Three architectural trends prevail in the latest ISIC 2018/HAM10000 literature (2022–2025). The first category comprises sophisticated convolutional ensembles, including DSCC_Net (94.17% accuracy on HAM10000) [20], the soft-attention CNN developed by Datta and associates (93.4%) [20], and SkinSage XAI (96% with AUC 0.998) [21]. The second category comprises pure or hybrid transformer designs, such as SkinFormer (Dice 0.932) [22], SapFormer (0.943) [23], DermViT [24], Swin-UNet [25], TransUNet [26], and the Bidirectional Attention Deformable U-Net (BiADATU-Net, Dice 0.91) [27]. The third involves multitask pipelines that concurrently segment and categorise lesions, with modifications of foundation models like MedSAM and SAM-Med2D. Notwithstanding these advancements, the methodological rigor and repeatability of the published figures exhibit significant variability; papers claiming accuracy above 96–98% on HAM10000 should be scrutinised for distinctions between image-level and lesion-level analyses, as well as potential duplicate-lesion leakage [13].

This study makes four contributions, all of them methodological rather than architectural. First, we construct a comparison of six classification configurations in which input resolution and pretraining corpus are held constant, so that the remaining variation is architecture family and capacity, and we analyse it at the level of the outer fold, which is the only genuinely blocked unit the design provides. Second, we separate the two prompting regimes under which a promptable segmentation foundation model can be evaluated and report both, so that the comparison against unprompted networks is like-efor-like. Third, we evaluate saliency localisation against a fixed, image-independent baseline, which is necessary because the mask-IoU metric has a high floor on centred dermoscopic lesions. Fourth, we report external validation on an independent cohort, in accordance with the TRIPOD + AI 2024 reporting guideline [28], with the sample underlying every column stated explicitly, and we distinguish development, selection and final estimation throughout. Explanations are computed with Grad-CAM, Grad-CAM++, Score-CAM and LIME, and calibration is reported before and after temperature scaling. The result is a set of weaker claims than those usually made in the literature. The advantage of the foundation segmentation model vanishes when its prompts are created without reference information, the six configurations cannot be statistically differentiated under this procedure, and the saliency–localisation measure is unable to differentiate the trained models from a fixed circle. Instead of reporting these results as a warning to a headline figure, we provide them as the main focus of this research. 

The subsequent sections of the paper are structured as follows. Section 2 delineates the dataset, ethical considerations, software and hardware infrastructure, preprocessing pipeline, augmentation strategy, model architectures, training regimen, assessment metrics, calibration methodology, explainable artificial intelligence techniques, and biostatistical analysis framework. Section 3 presents the outcomes of classification, segmentation, calibration, statistical analysis, and XAI. Section 4 examines the findings in relation to fifteen recent studies (2022–2025), addresses limitations and clinical implications, and delineates future directions, including foundational models, federated learning, and multimodal AI within the regulatory frameworks of the FDA Predetermined Change Control Plan (PCCP) and the EU AI Act. Section 5 concludes this study.

## 2. Materials and Methods

### 2.1. Dataset and Ethical Statement

We used the publicly available HAM10000/ISIC 2018 Challenge dataset, which comprises 10,015 dermoscopic images of 600 × 450 pixels distributed across seven diagnostic categories: melanocytic nevi (NV; *n* = 6705; 66.95%), melanoma (MEL; *n* = 1113; 11.11%), benign keratosis-like lesions (BKL; *n* = 1099; 10.97%), basal cell carcinoma (BCC; *n* = 514; 5.13%), actinic keratoses and intraepithelial carcinoma (AKIEC; *n* = 327; 3.27%), vascular lesions (VASC; *n* = 142; 1.42%), and dermatofibroma (DF; *n* = 115; 1.15%). Histopathology confirms more than half of cases, with the remaining determined by follow-up testing, expert consensus, or in vivo confocal microscopy. The ISIC 2018 challenge divided this collection into Task 1 (lesion border segmentation, 2594 training masks, 100 validations, 1000 test photos) and Task 3 (seven-class classification) [29].

The dataset is freely available in the ISIC Archive (https://challenge.isic-archive.com/data/#2018, accessed on 21 June 2026) and Harvard Dataverse (https://doi.org/10.7910/DVN/DBW86T, accessed on 21 June 2026) [30]. Figure 1 illustrates the class distribution.

Segmentation reference masks. Lesion masks for HAM10000 are not part of the original release. We used the mask set distributed by Tschandl and colleagues, which was generated by a neural network and subsequently corrected by a dermatologist. These masks are therefore semi-automatic and not expert-drawn, and every Dice, IoU and mask-IoU value computed against them in this paper should be read with that in mind. The expert-annotated ISIC 2018 Task 1 masks cover a different 2594 image subset and were not used.

External validation cohort. Cross-cohort generalisation was assessed on BCN20000 [31], acquired at the Melanoma Unit of the Hospital Clínic in Barcelona with a different dermatoscope, patient population and acquisition protocol from HAM10000, allowing us to test the geographic and acquisition domain shift in the cross-cohort validation design of Combalia et al. [32]. The HAM10000 images were accrued over a 20-year period at the two contributing centres [30], and the BCN20000 images between 2010 and 2016 at Hospital Clínic de Barcelona [31]; both are archival collections and no participant follow-up applies. The labelled release available at the time of this analysis contains 16,133 images across six of the seven HAM10000 categories; vascular lesions are absent, as are the squamous cell carcinoma and out-of-distribution categories, whose labels are released only for the challenge test split. The reported cross-cohort performance therefore does not describe the full BCN20000 spectrum. The cohort was divided into a 961-image calibration split, used for the single purpose of re-fitting the temperature, and a 15,172-image test split on which all performance metrics are computed; the two are disjointed and the calibration split contributes to no reported performance metric. No BCN20000 image contributed to training or hyperparameter selection.

A note on scope. An open-set analysis of the squamous cell carcinoma and out-of-distribution categories announced in the submitted version was not performed and is not reported, because the labels required for it are not part of the public release. The PH^2^ external segmentation cohort [33] is likewise not reported in this version; the evaluation has not been reproduced under the present protocol, and we prefer to omit it rather than carry forward a value we cannot verify.

Ethical statement. This work made use of the publicly accessible, completely de-identified HAM10000/ISIC 2018 Challenge dataset ADDIN EN.CITE ADDIN EN.CITE.DATA [29,30]. The institutional review boards of the Medical University of Vienna (protocol number 1804/2017) and the University of Queensland approved the original data collection and ethical procedure. Because the dataset is completely anonymised, includes no personally identifying information, and is freely available via the ISIC Archive and Harvard Dataverse under a Creative Commons license, no further ethical permission was necessary for the current secondary study. All methods involving personal data followed the guidelines of the Declaration of Helsinki (2013 version).

This study is reported in accordance with the TRIPOD + AI 2024 statement for the reporting of clinical prediction models developed or evaluated using artificial intelligence; the completed checklist is provided as Table S1 in the Supplementary Materials.

### 2.2. Software and Hardware

All experiments were implemented in Python 3.11 using PyTorch (version 2.13.0) [34] and MONAI [35]. Pretrained backbones were loaded from the timm library [36]. Image augmentation used Albumentations [37]; image processing primitives used scikit-image [38] and OpenCV. Classical machine learning utilities used scikit-learn [39]. Explainable AI methods used pytorch-grad-cam and the LIME library. Statistical analysis used SciPy [40] and statsmodels. Visualisation used Matplotlib (version 3.10.8) [41]. Exact package versions were pinned in the requirements file of the deposited archive (Data Availability Statement), which is the authoritative record. Training was performed on a single NVIDIA GeForce RTX 4050 Laptop GPU (6 GB) under Windows 11, using automatic mixed precision. All random seeds are fixed and recorded in the deposited archive.

### 2.3. Image Preprocessing Pipeline

The full preprocessing pipeline is shown in Figure 2, with representative dermoscopic examples illustrated in Figure 3. The pipeline comprises five sequential stages: hair removal, colour constancy, contrast enhancement, channel normalisation, and resizing.

#### 2.3.1. Hair Removal (DullRazor with Telea Inpainting)

Hair artefacts were detected by black-hat morphology on the grayscale image with a cross-shaped 17 × 17 structuring element, BH(I) = (I • B) − I, where • denotes the morphological closing operator. The detected hair mask was thresholded at an intensity τ = 10, dilated to ensure full-shaft coverage, and inpainted using the Telea fast-marching algorithm with a radius of 6 pixels [42]. This implementation reduces the hair saturation artefact without erasing pigment network detail.

#### 2.3.2. Colour Constancy (Shades-of-Gray)

Inter-device chromatic variability was reduced using the Shades-of-Gray algorithm with the Minkowski norm exponent *p* = 6:

(1/*N* · *Σ_x_ I^c^*(*x*)*^p^*)^1/*p*^ = *k* · *e^c^*,    *c* ∈ {R, G, B} (1)

where *e^c^* is the per-channel illuminant estimate. Each channel was rescaled by the inverse of its normalised illuminant. This step alone is reported to increase melanoma sensitivity from 71% to 79.7% and specificity from 55% to 76% on classical ISIC benchmarks [12].

#### 2.3.3. Contrast Enhancement (CLAHE on L*)

Each image was converted from RGB to the perceptually uniform CIE L*a*b* colour space; CLAHE (clipLimit = 2.0, tileGridSize = 8 × 8) was applied to the L* (luminance) channel only [43] and the channels were merged before re-conversion to RGB. Operating on L* avoids the chromatic distortion that arises when CLAHE is applied to each RGB channel independently.

#### 2.3.4. Resizing and Normalisation

Images from all seven diagnostic categories (Table 1) were resized by bicubic interpolation to 224 × 224 pixels for classification and 256 × 256 for segmentation. The classification resolution is identical for every architecture. The submitted version used 384 × 384 for three of the twelve models and 224 × 224 for the remainder, which confounded architecture with input resolution; this is corrected here. Per-channel z-score normalisation used canonical ImageNet statistics: mean = [0.485, 0.456, 0.406] and standard deviation = [0.229, 0.224, 0.225]. The complete preprocessing parameter set is reported in Table 2.

### 2.4. Data Augmentation and Class Imbalance Handling

Geometric augmentation included horizontal flip (*p* = 0.5), vertical flip (*p* = 0.5), rotation in the range ±180° (*p* = 0.7), affine transforms, and elastic transforms (α = 120, σ = 6). Photometric augmentation included HueSaturationValue (10, 15, 10), RandomBrightnessContrast (0.2, 0.2), and RandomGamma (80, 120). Advanced policies included RandAugment (*N* = 2, *M* = 9), MixUp [44], and CutMix [45]. CoarseDropout used eight square holes of 32 × 32 pixels. 

MixUp linearly interpolates two image–label pairs (*x*_i_, *y*_i_) and (*x*_j_, *y*_j_):

(2) $$\tilde{x}=\lambda x_{i}+(1-\lambda )x_{j}$$

ỹ = λy_i_ + (1 − λ)y_j_,    λ∼Beta(0.2, 0.2) (3)

CutMix replaces a rectangular region of one image with a patch from another:

(4) $$\tilde{x}=M⊙x\text{\_}A+(1-M)⊙x\text{\_}B$$

ỹ = λy_A + (1 − λ)y_B,    λ∼Beta(1.0, 1.0)
(5)

where M ∈ {0, 1}^(H × W) is a binary patch mask whose area equals (1 − λ) · *H* · *W*, and ⊙ denotes element-wise (Hadamard) multiplication.

To address the severe class imbalance shown in Figure 1 (NV = 66.95% versus DF = 1.15%), we combined a weighted random sampler with sample weights,

*w*_i_ = 1/*n*_class(*i*), (6)

and with the class-balanced focal loss [46]

*L*_FL = −α_c (1 − *p*_c)^γlogp_c, (7)

using γ = 2 and per-class α_c set to inverse class frequency. For segmentation, a single loss is used throughout, L = 0.5·L_BCE + 0.5·L_Dice (Section 2.7). The submitted version specified a focal–Tversky loss at this point and the Binary Cross-Entropy plus Dice loss in Section 2.7; the two statements were inconsistent, and the focal–Tversky specification has been removed. The implementation in the deposited archive matches the formula given in Section 2.7 and can be checked against it.

*L*_FT = (1 − TI)^(1/γ), TI = TP/(TP + α·FN + β · FP) (8)

with α = 0.3, β = 0.7, and γ = 4/3, up-weighting false negatives to address lesion-versus-background imbalance.

### 2.5. Stratified Group Splitting

A critical preliminary observation is that HAM10000 contains only 7470 unique lesions for its 10,015 images: a single physical lesion may have been imaged at multiple time points or magnifications [13]. Random image-level splitting therefore inflates apparent test accuracy by 1–3 percentage points through near-duplicate leakage. We accordingly used scikit-learn StratifiedGroupKFold (n_splits = 5) with the lesion_id as the grouping variable, ensuring that all images of any given lesion appear in the same fold.

Inner-validation partition. Within each of the five outer folds, the four training folds are further divided at the lesion level into a 90% training and a 10% inner-validation partition. Early stopping (patience 15 epochs on inner-validation balanced accuracy) and the temperature-scaling fit both use this inner-validation partition and nothing else; the outer test fold contributes to neither. The submitted version referred to a “held-out validation set” that was never defined, leaving the reader unable to establish whether the temperature had been fitted on the test data. The fold assignment file in the deposited archive allows the partition to be reconstructed image by image.

Repetitions. One seed is used per fold. The submitted version used three seeds per fold and treated the resulting 5 × 3 = 15 values as independent paired observations. They are not: seeds within a fold share the same partition and contribute no additional blocking. The unit of analysis is therefore the outer fold, N = 5 (Section 2.11).

External cohort. BCN20000 contributed no image to training or hyperparameter selection. The disjointed 961-image calibration split was used for one purpose only, re-fitting the temperature, and is excluded from every reported performance metric; the sample underlying each reported result is stated in the corresponding table caption. The submitted version presented in Section 2.1 claimed that the external cohorts contributed nothing to the temperature-scaling fit while stating that a subset had been reserved for exactly that purpose. Both could not hold, and the design is now stated once, consistently.

### 2.6. Deep Learning Models for Classification

Six architectures were benchmarked, spanning convolutional and transformer families: ResNet-50 (23.5 M parameters) [47], DenseNet-121 (7.0 M) [47], EfficientNet-B3 (10.7 M) [47], ConvNeXt-T (27.8 M) [48], Swin-T (27.5 M) [49], and ViT-B/16 (85.8 M) [50]. Design controls. Every configuration receives 224 × 224 input and ImageNet-1k pretraining. These two factors are constant across the comparison and cannot explain any difference observed between models. This is a change from the submitted version, in which resolution varied between 224 and 384 and the pretraining corpus between ImageNet-1k and ImageNet-21k, both in the direction that favoured the model subsequently reported as best. What remains uncontrolled is parameter count, which spans 7.0 M to 85.8 M and cannot be equalised across architecture families without distorting each family’s design; the comparison is accordingly described throughout as one of complete configurations, not of architectures in isolation. Reduction of the model set. The classifier set was reduced from twelve to six. Taking a maximum of twelve candidates evaluated on a single cross-validation framework is upward-biased, and a smaller pre-specified set reduces the number of comparisons from which an apparent winner can emerge. The six retained models were chosen to span the convolutional–transformer axis and the capacity range, before any results were examined. All were fine-tuned with AdamW under a OneCycle cosine schedule, an initial learning rate of 1 × 10^−3^ for convolutional and 1 × 10^−4^ for transformer backbones, weight decay 1 × 10^−4^ and 0.05, respectively, a batch size of 16–24 with gradient accumulation, a maximum of 60 epochs, label smoothing of 0.1, and mixed precision. Full hyperparameters are given in Table 3.

### 2.7. Deep Learning Models for Segmentation

Five segmentation architectures were benchmarked: U-Net (24.4 M) [51], U-Net++ (26.1 M) [52], Attention U-Net (24.6 M) [53], DeepLabV3+ (26.7 M) [54], and SegFormer-B2 (27.3 M) [55]. All were optimised with AdamW on the combined loss L = 0.5·L_BCE + 0.5·L_Dice at 256 × 256 resolution. MedSAM (ViT-B backbone, 93.7 M) [56] was evaluated separately, for the reason given below. Training hyperparameters are summarised in Table 3. Prompting regimes. MedSAM is a promptable model and its performance depends on where the prompt comes from. The submitted version described prompts “derived from the union of pseudo-prompted lesion bounding boxes” without defining the term, and then placed the resulting score in the same table as networks receiving no localisation information at all. That comparison was not like for like. We therefore evaluate MedSAM under two explicitly separated regimes and report both. Under reference-derived box prompts, the bounding box is computed from the reference mask with 5% random jitter; this uses information that would not be available at deployment and constitutes an upper bound, not comparable with the unprompted networks, and it is labelled as such in the segmentation results. Under fully automatic box prompts, the bounding box is produced from the image alone by Otsu thresholding followed by largest-connected-component selection, using no reference information whatsoever; this is the only regime in which MedSAM and the unprompted networks measure the same thing, and it is the regime on which any deployment-facing claim must rest.

### 2.8. Evaluation Metrics

#### 2.8.1. Classification Metrics

We computed the accuracy, sensitivity (recall), specificity, precision (positive predictive value), F1-score (macro), Matthews Correlation Coefficient (MCC), balanced accuracy, ROC-AUC (one-versus-rest macro), PR-AUC, Brier score, and Cohen’s κ. The MCC, which is robust to class imbalance, is defined as MCC = (TP·TN − FP·FN)/√((TP + FP)(TP + FN)(TN + FP)(TN + FN)) [57,58]. The Brier score, BS = (1/N) Σ (p_i_ − y_i_)^2^, is reported as a joint measure of discrimination and calibration. The multi-class MCC follows Gorodkin’s K-category extension [59]. Majority-class baseline. Because 66.95% of the development cohort belongs to a single class, accuracy in this setting has a floor near 0.67 for a classifier that learns nothing. We therefore report a majority-class baseline row in the internal and external classification results (Section 3.1 and Section 3.8), and we treat balanced accuracy, whose floor is 1/7 = 0.143 internally and 1/6 = 0.167 externally, as the primary metric. Accuracy alone should not be read in this cohort.

#### 2.8.2. Segmentation Metrics

Segmentation performance was reported using the Dice Similarity Coefficient (DSC = 2|A ∩ B|/(|A| + |B|)), Intersection over Union (IoU = |A ∩ B|/|A ∪ B|), the 95th percentile Hausdorff Distance (HD95), and Average Surface Distance (ASD). Aligned with the Metrics Reloaded recommendations [60], we report multiple complementary metrics rather than relying on Dice alone. All values are computed against the semi-automatic reference masks described in Section 2.1.

### 2.9. Calibration Analysis

Reliability diagrams used 15 equal-width confidence bins. The expected calibration error was computed as ECE = Σ_m (|B_m|/N) · |acc(B_m) − conf(B_m)|, with sensitivity to bin count verified at 10 and 20 bins, per the recommendations of Nixon and colleagues [61]. We additionally report the Maximum Calibration Error (MCE) and the Adaptive Calibration Error (ACE) using equal-mass binning. Post hoc temperature scaling was applied as $\hat{p}$ = softmax(z/T), with T optimised via L-BFGS minimisation of the negative log-likelihood [15]. We confirmed that argmax accuracy was unchanged after calibration. The temperature was fitted on the inner-validation partition of the same fold, as defined in Section 2.5, and never on the outer test fold on which the calibration metrics were then measured.

### 2.10. Explainable Artificial Intelligence

Explanations were computed for the configuration with the highest mean-fold balanced accuracy, on a stratified 300-image subset of the held-out data. Three class activation methods were applied—Grad-CAM applied to the last convolutional block, with L^c^ = ReLU(Σ_k_ α_k_^c^ A^k^) [62]; Grad-CAM++ for multifocal lesions [63]; and Score-CAM as a gradient-free check [64]—together with LIME using SLIC superpixels [65]. Maps were computed for the predicted class, which is not always the true class; the figures state the prediction and its probability on every panel. Localisation baseline. Saliency localisation was quantified by mask-IoU: the upper quintile of the saliency mass was binarised and intersected with the reference lesion mask. This metric has a high floor on dermoscopic images, because lesions are large and approximately centred, so an absolute value is uninterpretable on its own. We therefore compute the mask-IoU obtained by a fixed, image-independent centred disc whose radius is set from the mean lesion area across the cohort, and report every saliency score as a margin over that baseline. The submitted version reported mask-IoU values without such a reference and concluded from them that the model attends to lesion content; that inference does not follow, and Section 3.6 reports what the baseline comparison shows. Radiomic attribution analysis. A secondary analysis examines which image-derived quantities track the model’s melanoma probability. Twelve features are computed by us directly from the preprocessed image and the predicted lesion mask: two asymmetry indices about the principal axes, border irregularity (perimeter^2^/4π·area), lesion area and equivalent diameter, per-channel colour means and standard deviations in HSV, a colour variegation index, and grey-level co-occurrence contrast, homogeneity and energy. Every feature is computed from the image; none is an expert annotation and none is imported from an external annotated cohort. The submitted version listed “blue-white veil” and “pigment network atypia” among the features; these are interpretive dermoscopic criteria requiring expert reading, they are not available as annotations in HAM10000, and they have been removed. A logistic regression over the CNN melanoma logit plus the twelve features is fitted under five-fold cross-validation on 299 images (33 melanomas), and attributions are computed with TreeSHAP [66,67]. We report the cross-validated discrimination only; the apparent resubstitution value is a memorisation artefact of fitting 13 predictors to 299 observations and is not reported as a performance estimate.

### 2.11. Biostatistical Analysis

Unit of analysis. Configurations are compared across the five outer folds, N = 5 blocks and k = 6 treatments. The fold is the only genuinely blocked unit that this design provides. The submitted version used 5 folds × 3 seeds = 15 paired observations; these are not independent, because seeds within a fold share a partition, and treating them as such inflates apparent significance. Multi-configuration comparison used the Friedman test followed, only if the omnibus test rejects, by the Nemenyi post hoc procedure with a critical-difference diagram [68], CD = q_α √(k(k + 1)/6N). Bootstrap BCa 95% confidence intervals used 2000 resamples via scipy.stats.bootstrap. Where pairwise tests are reported, they are Holm–Bonferroni-corrected [69,70]. A residual dependence we do not correct. Even at the fold level, the five observations are not strictly independent, since any two training folds share three-quarters of their images. This positive correlation tends to make such tests anticonservative—that is, too ready to reject. We state this rather than leave it to be inferred. As reported in Section 3.5, the omnibus test does not reject even so, and the conclusion is therefore robust to the residual dependence: a test biased towards finding differences did not find one. Development, selection and estimation. We distinguish three stages throughout. Development is the cross-validated fitting on HAM10000. Selection is the choice of a single configuration on the basis of that same evaluation, which is upward-biased and which, as Section 3.5 shows, the statistics do not license in this study. Final estimation is the external evaluation on BCN20000, which did not inform selection and is the quantity that bears on generalisation.

Table 1 details the exact seven-class composition of the dataset, listing the image count, relative frequency, and ground-truth source for each diagnostic category.

Figure 2 provides a schematic overview of the complete end-to-end workflow, tracing an input dermoscopic image through preprocessing, segmentation, classification, calibration, statistical analysis, and explainability.

Figure 3 illustrates the sequential effect of each preprocessing stage on a representative dermoscopic image, together with examples of the geometric and mixing augmentations applied during training.

Table 2 specifies the full set of preprocessing and augmentation parameters, with the corresponding library and version for each step to support exact reproducibility.

Table 3 summarises the training hyperparameters for each model group, including optimiser, learning rate schedule, batch size, epochs, and weight decay.

## 3. Results

### 3.1. Classification Performance

Table 4 reports the performance of the six configurations, averaged over the five held-out outer folds with bootstrap BCa 95% confidence intervals, after temperature scaling. Balanced accuracy spans a range of 3.6 percentage points, from 0.664 (ViT-B/16) to 0.700 (ConvNeXt-T), with EfficientNet-B3 at 0.691, Swin-T at 0.690, ResNet-50 at 0.686 and DenseNet-121 at 0.668. The confidence intervals overlap throughout: the interval for the top configuration, [0.674, 0.714], contains the point estimate of four of the other five. Discrimination is considerably stronger than the threshold-dependent metrics suggest, with ROC-AUC between 0.933 and 0.950 for all six; macro-F1 lies between 0.585 and 0.640 and MCC between 0.540 and 0.607. The gap between AUC and macro-F1 reflects the severe class imbalance: the models rank lesions well but allocate the rare classes poorly at the argmax threshold. The majority-class baseline. A classifier that assigns every image to melanocytic nevi achieves an accuracy of 0.669 in this cohort. Four of the six configurations report accuracy between 0.709 and 0.732, that is, between four and six points above a model that has learned nothing. This is why balanced accuracy, for which the same baseline scores 0.143, is treated as the primary metric. Relation to the previously reported values. The submitted version reported an accuracy of 0.918 and a balanced accuracy of 0.870 for ConvNeXt-B. Those values are not reproduced here and are withdrawn. The present figures are what this benchmark yields under lesion-level grouping with no selection over a large candidate set, and the direction and rough magnitude of the discrepancy are consistent with the leakage and selection effects documented by Cassidy and colleagues [13]. Normalised confusion matrices are presented in Figure 4 and ROC and precision–recall curves in Figure 5.

### 3.2. Segmentation Performance

Table 5 reports segmentation performance on the held-out fold against the semi-automatic reference masks. The five unprompted networks are tightly grouped: SegFormer-B2 attains Dice of 0.9416, U-Net++ 0.9406, U-Net 0.9402, Attention U-Net 0.9401 and DeepLabV3+ 0.9332. The spread across five architectures of differing design is 0.8 Dice points. MedSAM under the two prompting regimes. With reference-derived box prompts, MedSAM attains Dice of 0.9547, IoU of 0.9147 and HD95 of 6.38 px, the best values in the table. With fully automatic box prompts, on the same images and the same weights, it attains Dice of 0.7729, IoU of 0.7019 and HD95 of 35.29 px—last place, 16.7 Dice points below the plain U-Net, with a boundary error more than five times larger. The difference between the two regimes is 18.2 Dice points, more than twenty times the spread across all five unprompted architectures. What was previously being measured was therefore the quality of the prompt rather than the quality of the model, and the claim in the submitted version that MedSAM significantly outperformed every non-foundation baseline is withdrawn. Figure 6 shows qualitative overlays for four cases sampled across the Dice distribution, with per-panel values between 0.937 and 0.985.

### 3.3. Learning Behaviour

All six configurations converged under the OneCycle schedule within the 60-epoch budget, with early stopping triggered on inner-validation balanced accuracy. We report convergence as a descriptive observation only and draw no inference from the learning behaviour; in particular, we make no claim about over-fitting, since the quantity that bears on that question is the held-out fold performance already reported in Table 4.

### 3.4. Calibration

Before temperature scaling, all six configurations were over-confident, a pattern characteristic of modern deep networks [15]. Expected calibration error ranged from 0.0405 (DenseNet-121) to 0.0774 (ConvNeXt-T). After temperature scaling fitted on the inner-validation partition, ECE fell for every configuration, to a range of 0.0273 to 0.0382, with argmax accuracy unchanged; for ConvNeXt-T, the values are 0.0774 before and 0.0274 after, at T = 0.757. The fitted temperatures lie between 0.757 and 0.967 and are reported per configuration in Table 4. All six are below 1.0, which corresponds to sharpening rather than softening the predictive distribution—the opposite of the pattern reported in the submitted version, where a temperature of 1.83 was given, and resulting in the lower absolute confidence of models trained under a corrected protocol. Reliability diagrams before and after scaling are shown in Figure 7.

### 3.5. Statistical Comparisons

The principal result of this study is reported here. Across the five outer folds and six configurations, the Friedman omnibus test yields χ^2^ = 7.057 with *p* = 0.216. The null hypothesis of equal performance is not rejected. With a critical difference of 3.3722 at α = 0.05, the equivalence cluster spans the entire model set: ConvNeXt-T, EfficientNet-B3, Swin-T, DenseNet-121, ResNet-50 and ViT-B/16 (Table 6, Figure 8). No post hoc pairwise comparison is therefore performed, and none is reported. No configuration is designated best anywhere in this manuscript. A 3.6-point spread in balanced accuracy across six architectures spanning convolutional and transformer families, and a twelve-fold range of parameter count, is not distinguishable from fold-to-fold variation at N = 5. We emphasise that this is a failure to reject, not a demonstration of equality; five blocks provide limited power, and a larger study might separate these models. What can be said is that the differences routinely reported in the literature, which are of this magnitude or smaller, are not supported by the evidence typically presented for them. As noted in Section 2.11, the residual positive correlation between folds biases the test towards rejection, so the failure to reject is unlikely to be an artefact of the dependence structure. What was previously reported. The submitted version reported a Friedman statistic of 47.6 with *p* < 0.001 across 12 classifiers and 15 paired observations, together with McNemar and DeLong tests establishing pairwise superiority and a claim that MobileNetV3-L was significantly inferior. Those analyses rested on the seed replication counting described in Section 2.11 and are withdrawn in their entirety.

### 3.6. Preprocessing Components Were Not Ablated

The preprocessing pipeline was applied as a fixed block in this study and its components were not ablated. A single-fold exploratory run was performed but not repeated across all five folds, and a one-fold difference cannot be interpreted without between-fold variance—particularly given that the six configurations reported here span only 3.6 balanced-accuracy points and are statistically indistinguishable (Section 3.5). The ablation table of the submitted version and the per-component contribution figures attached to it are therefore withdrawn; the implications for what this paper can claim are stated in Section 4.4.

### 3.7. Explainable AI

Radiomic attribution. Under five-fold cross-validation, the logistic regression over the CNN melanoma logit plus twelve image-derived features attains an AUC of 0.9118. The CNN logit alone attains 0.9158. The twelve radiomic features therefore contribute −0.0040—nothing, and marginally worse than nothing. TreeSHAP is consistent with this: the CNN logit has a mean absolute SHAP value of 1.99 and border irregularity of 0.49, and the remaining features are negligible (Figure 9). The submitted version presented this analysis as a fusion model whose feature ranking aligned with the ABCDE rule; it is reframed here as an interpretability probe with no performance contribution, and the alignment claim is not made. The sample is 299 images (33 melanomas), not the 320 stated previously.

Figure 10 shows LIME attributions for four held-out cases, one per diagnostic class, selected by a fixed rule that does not consider whether the prediction was correct: green superpixels contribute positively to the predicted class and red negatively. In two of the four cases, the lesion core itself carries negative weight while the supporting superpixels lie in perilesional skin. We report this without interpretation; it is consistent with the localisation result below, and neither observation supports a claim that the explanations correspond to dermoscopic criteria. The LIME stability figure reported in the submitted version has not been reproduced under the present protocol and is withdrawn.

Table 7 reports the mask-IoU for the three class activation methods together with the fixed centred-disc baseline. The baseline, which uses no information from the image, scores 0.4745 on the 300-image explainability subset (0.4898 across all 10,015 masks). Grad-CAM++ scores 0.3512, Score-CAM 0.3351 and Grad-CAM 0.2531. All three methods fall below the trivial baseline, by margins of 0.123, 0.139 and 0.221, respectively. The metric therefore does not distinguish learned attribution from a fixed circle on this task, and no conclusion about lesion-focused attention can be drawn from values in this range. The statement in the submitted version that the model bases its predictions on lesion content rather than peri-lesion artefacts—described there as a critical finding for clinical credibility—is withdrawn. It did not follow from the evidence, and the evidence now available points the other way. Figure 11 shows the same result case by case. Per-panel mask-IoU ranges from 0.037 to 0.374 across twelve panels, with none reaching the baseline. In the actinic keratosis panel, Score-CAM places its strongest activation in the image corners, outside the lesion entirely, yielding a mask-IoU of 0.000. Two of the four displayed cases are misclassified, and the maps for those cases explain an incorrect decision, which the figure states.

The six-method Quantus panel reported in the submitted version has not been reproduced under the present protocol and is omitted, together with its table and heatmap. We prefer to omit values we cannot currently verify rather than carry them forward.

Figure 6 provides qualitative segmentation overlays for four representative lesions, showing that residual errors concentrate at the lesion border rather than reflecting systematic mis-localisation.

Figure 7 contrasts the reliability diagrams before and after temperature scaling, demonstrating that calibration sharply reduces over-confidence without altering argmax accuracy.

### 3.8. External Validation on BCN20000

Table 8 reports performance on the 15,172-image BCN20000 test split, with no fine-tuning and no cohort-specific adaptation of any kind for every column except the last. The HAM10000-fitted preprocessing pipeline was applied unchanged. These are the weakest results in this paper and we report them without softening. Balanced accuracy falls by a mean of 25 percentage points. Internal values of 0.664 to 0.700 become external values of 0.401 to 0.476, with individual drops ranging from −21.4 points (Swin-T) to −27.5 points (EfficientNet-B3). The internal ranking reverses. ConvNeXt-T leads internally at 0.700 but is second externally at 0.451; Swin-T is fourth internally at 0.690 and first externally at 0.476. A ranking that does not survive a change of cohort was not estimating a stable property of the architectures, and this is direct evidence for the selection optimism argument set out in Section 2.11. Discrimination transfers considerably better than allocation. External ROC-AUC remains between 0.8015 and 0.8401 while balanced accuracy collapses. The class distribution differs markedly between the two cohorts, and it is the decision threshold rather than the ranking ability that fails to transfer. The majority-class baseline on this cohort achieves an accuracy of 0.3528, against reported accuracies of 0.518 to 0.554. The evaluation covers six of the seven HAM10000 classes; vascular lesions, squamous cell carcinoma and out-of-distribution lesions are absent from the labelled release, so these figures do not describe the full BCN20000 spectrum.

Calibration under domain shift. With the HAM10000-fitted temperature and no adaptation, external ECE ranges from 0.0286 to 0.0576. After re-fitting the temperature on the disjointed 961-image calibration split, it ranges from 0.0371 to 0.0459. Local re-fitting improves calibration for two configurations (DenseNet-121, 0.0576 → 0.0394; ConvNeXt-T, 0.0546 → 0.0459) and worsens it for two others (ResNet-50, 0.0286 → 0.0371; EfficientNet-B3, 0.0407 → 0.0434). A single global temperature is too coarse an instrument to absorb a shift of this kind. The submitted version reported that a cohort-specific re-fit restored calibration to 0.025 and described the cross-cohort gap as modest and recoverable; neither statement is supported by the present results. The clinical consequence is set out in Section 4.5.

External segmentation validation on PH^2^ is not reported in this version. The evaluation has not been reproduced under the present protocol, and we prefer to omit it rather than carry forward an unverified value; the implication for the scope of the external-validity claim is stated in Section 4.4.

Taken together, the external results show a large rather than modest cross-cohort gap, a ranking that does not transfer, and a calibration loss that a single-parameter local correction does not reliably repair.

## 4. Discussion

### 4.1. Principal Findings

This study set out to measure how much of the reported performance of a dermoscopic deep learning pipeline survives an evaluation protocol designed to remove the sources of optimism. The answer is as follows: less than the literature, including our own submitted version, would suggest. Four findings carry this paper. First, when input resolution and pretraining corpus are held constant and the analysis is conducted at the level of the outer fold, six architectures spanning convolutional and transformer families and a twelve-fold range of parameter count cannot be separated statistically (χ^2^ = 7.057, *p* = 0.216). Second, the advantage of a promptable segmentation foundation model is entirely attributable to prompt provenance: MedSAM ranks first with reference-derived prompts and last with automatic prompts, an 18.2-point Dice difference against a 0.8-point spread across five unprompted architectures. Third, a fixed circle outperforms three class activation methods on the mask-IoU metric; so, this metric cannot support claims about lesion-focused attention. Fourth, cross-cohort transfer costs 25 points of balanced accuracy and reverses the internal ranking, and local recalibration does not reliably repair the calibration loss. Each of these is a negative result, and we present them as the substance of the work rather than as limitations attached to a headline number. Their common structure is that a comparison was previously being made between things that differed in more than the factor named, and that the apparent effect disappeared once the other factors were held fixed. We would suggest that this structure is not specific to our study. On the mask-IoU baseline in particular. The most transferable finding here may be the smallest one. Mask-IoU has a high floor on dermoscopic images because lesions are large and approximately centred; a fixed disc scores 0.4745. Values in the 0.4–0.5 range have been reported in the literature as evidence of lesion-focused attention. They are not evidence of anything unless a matched trivial baseline is reported alongside them, in the spirit of the sanity checks proposed by Adebayo et al. [71], and the cost of computing one is negligible.

### 4.2. Comparison with Recent Literature

Cassidy and colleagues [13] performed a systematic methodological audit of ISIC dataset usage and recommended explicit lesion-level group splitting and standardised reporting; our pipeline implements both. Their finding that image-level splits inflate apparent accuracy is directly relevant to the comparison between our present and previously reported figures. The headline numbers in the recent HAM10000 classification literature—DSCC_Net at 94.17% [20], the soft-attention CNN of Datta and colleagues at 93.4% [72], the wavelet–EfficientNet hybrid of Aboulmira and colleagues at 94.7% [73], the two-stage voting ensemble of Chiu and colleagues at approximately 96% [74], and SkinSage XAI at 96% with AUC 0.998 [21]—are far above the values we report. We do not claim that these results are wrong. We observe that they are not directly comparable with ours, because most are obtained under image-level rather than lesion-level splitting, several on subsets of the cohort, and because the reported figure is in each case a maximum over a candidate set evaluated on the same data. Our own submitted version reported 0.918 under conditions closer to theirs than to the present conditions, and the difference between 0.918 and 0.770 is a measure of how much protocol choices matter on this benchmark. The Federated Skin Lesion classification work of Haggenmüller and colleagues [75] confirms that decentralised training across multiple centres can match centralised performance. Our pipeline is complementary to—rather than competing with—the headline accuracy literature in that it reports what the same benchmark yields when the comparison is constructed to isolate the factor named.

In segmentation, the reported Dice band on ISIC 2018—SapFormer 0.943 [23], SkinFormer 0.932 [22], ScaleFusionNet 0.918 [76], BiADATU-Net approximately 0.91 [27], and Swin-UNet 0.895 [25]—brackets the values obtained by our unprompted networks (0.933–0.942). What our results add is the observation that where such a band contains a promptable foundation model, the prompting regime needs to be stated before the comparison can be read: a model evaluated with reference-derived prompts and one evaluated end to end are not measuring the same quantity, and the difference in our hands is larger than the entire spread of the band. The DermViT diagnosis-guided transformer of Zhang and colleagues [24] and the SLICE-3D dataset released with ISIC 2024 [77] indicate directions this work does not cover. PanDerm (Yan and colleagues, Nature Medicine 2025) [78] is pretrained on over two million images from eleven institutions across four modalities including dermoscopy and clinical photography, and is evaluated across twenty-eight benchmarks covering diagnosis and segmentation. Whether a domain-pretrained backbone of this kind would break the equivalence cluster reported in Table 6 is an open question that our results cannot answer, and which we regard as the most interesting thing to test next.

### 4.3. Strengths

The strengths of this work are procedural rather than architectural. (i) Input resolution and pretraining corpus are held constant across the architecture comparison, so the comparison is of complete configurations under matched conditions. (ii) The two prompting regimes of the foundation segmentation model are separated and both are reported. (iii) Saliency localisation is evaluated against a fixed, image-independent baseline. (iv) The unit of statistical analysis is the outer fold, and correlated repetitions are not counted as independent observations. (v) The temperature-scaling partition is defined explicitly and is never the test fold. (vi) Lesion-level StratifiedGroupKFold prevents duplicate-lesion leakage. (vii) The sample underlying every column of the external table is stated in its caption. (viii) Code, seeds, fold assignments, weights and per-fold logs are deposited under a DOI, so that every table and figure can be regenerated from the deposit. The submitted version listed a controlled preprocessing ablation quantifying the contribution of each module and described the pipeline as an end-to-end, multi-task system explicitly coupling preprocessing, segmentation and classification. Neither claim is made here. The ablation is discussed in Section 4.4. The two branches are independent: segmentation output is not an input to classification, they share only a preprocessing front end and an evaluation protocol, and Figure 2 has been redrawn to show them as parallel paths. The single place where a segmentation output enters another computation is the radiomic analysis of Section 2.10, where the predicted mask defines the region over which features are computed; that is a post hoc analysis of the classifier’s output, not a coupling of the two branches.

### 4.4. Limitations

Preprocessing components were not ablated. The preprocessing pipeline was applied as a fixed block. A single-fold exploratory run was performed but not repeated across all five folds, and we judged a one-fold difference uninterpretable in the absence of between-fold variance, particularly given that the six configurations reported here span only 3.6 balanced maccuracy points and are statistically indistinguishable. We therefore make no claim about the individual contribution of hair removal, colour constancy, contrast enhancement or the augmentation policy. Whether any of these steps is necessary, redundant or harmful on this benchmark remains open, and a properly replicated component ablation is a natural target for future work. Parameter count is not controlled. Resolution and pretraining are held constant but capacity is not, ranging from 7.0 M to 85.8 M; it cannot be equalised across architecture families without distorting each family’s design. Capacity does not order the results—the largest model ranks last and the second-smallest ranks second—but this is an observation, not a control. Statistical power. Five blocks provide limited power, and the failure to reject is not a demonstration of equality. The direction of the residual fold dependence works against us rather than for us (Section 2.11), but a larger replication could separate configurations that we cannot. Reference masks are semi-automatic. All Dice, IoU and mask-IoU values are computed against network-generated, dermatologist-corrected masks. They are not an expert-drawn reference, and both the segmentation scores and the saliency baseline inherit whatever systematic bias that reference carries.

External validation is limited in extent and in kind. One external cohort is reported, covering six of seven classes, and no external segmentation cohort is reported in this version. Both development and external cohorts are European, specialist-centre and dermoscopic. Fitzpatrick phototype is not recorded in a form permitting stratified analysis, so no fairness evaluation was possible; Daneshjou and colleagues [79] have shown substantial performance disparities across skin tones on a diverse curated clinical dataset, and nothing in our results speaks to that question. Our findings may not generalise to all skin tones until evaluated on multi-Fitzpatrick cohorts such as Diverse Dermatology Images or Fitzpatrick17k.

Spectrum and prevalence. The development cohort was assembled at specialist centres from lesions already selected for imaging, with diagnoses established by histopathology, follow-up, expert consensus or confocal microscopy. Prevalence and case mix in an unselected primary-care population differ substantially and positive predictive value would fall accordingly. The 25-point drop we observe between two curated specialist cohorts sets a lower bound on what should be expected from a further move to an unselected population.

No clinician read these images. No reader study was conducted. The accuracy reported here cannot be placed against dermatologist or general-practitioner performance on the same cases, and we make no such comparison. For the same reason, we make no claim that the explanations in Section 3.7 help clinicians detect model errors or calibrate trust; establishing that would require a prospective reader study.

Melanoma is not a homogeneous label. HAM10000 provides no melanoma subtype annotation, so subtype-stratified performance could not be examined. Since dermoscopic criteria and their reliability differ across subtypes [8], heterogeneity within the melanoma class is an unmeasured source of variation in every figure we report.

Further limitations carried over from the submitted version: per-class sensitivity for the rarest categories remains the principal residual weakness despite focal-loss compensation and weighted sampling, a gap that may be addressable through diffusion model synthetic augmentation [80]; the experiments use dermoscopic imagery only and do not include smartphone clinical photographs, which represent the dominant input modality in primary-care telemedicine; the design is retrospective, and prospective clinical evaluation is required before any operational deployment claim; and patient metadata were not fused. We previously stated that metadata fusion is consistently shown to add 1–2 percentage points; a dataset descriptor does not establish a consistent effect size across studies, and that claim has been removed.

### 4.5. Clinical Implications

The submitted version proposed that a pipeline of this kind could stratify lesions in primary care or teledermatology into “biopsy now”, “refer to dermatology”, “monitor” and “reassure”. That proposal does not follow from the evidence presented here and is withdrawn, together with the accompanying statement about the number of benign biopsies per malignant lesion in primary care, for which we could identify no source supporting a single figure across settings. What the present results do bear on is narrower. A model of this kind should not be described as plug-and-play. Deployment at a new site should be assumed to require locally labelled data for recalibration, and our results indicate that even then a single global temperature may not suffice, since local re-fitting worsened calibration for two of six configurations. The threshold-dependent performance on which a triage pathway would depend is precisely the part that transfers worst, while ranking ability transfers comparatively well; a system built on this evidence would be better conceived as a ranking or prioritisation aid under local threshold calibration than as an autonomous four-way classifier. The DermaSensor device, the first FDA-authorised standalone AI-enabled skin cancer detection device for primary care [6,7], provides a useful reference point for what a regulator has been willing to accept: high sensitivity purchased at a specificity of 20.7%, with a number needed to biopsy of six in its pivotal study. The relevant comparison for academic work is not headline accuracy but the operating point and the population in which it was measured. In a second-reader role, calibrated systems can raise sensitivity without sacrificing specificity, in concordance with the human–computer collaboration evidence reported by Tschandl and colleagues [81]. Distribution shift, demographic equity, workflow integration and post-market surveillance remain operational challenges that academic accuracy benchmarks alone cannot resolve.

### 4.6. Future Directions

Four directions follow directly from the negative results reported here: first, a properly replicated preprocessing ablation, at five folds, to settle whether the components of the standard dermoscopic preprocessing block earn their place; second, evaluation of a domain-pretrained backbone such as PanDerm [78] under the present protocol to test whether large-scale dermatological pretraining separates configurations that ImageNet pretraining does not; third, saliency–localisation metrics with matched trivial baselines reported as standard and, more ambitiously, localisation references that reflect dermoscopic criteria rather than lesion extent, since a map that covers the lesion is not thereby a map that uses diagnostic features; fourth, a prospective reader study, without which no claim about the clinical utility of explanations can be made. Beyond these, the broader agenda remains as previously stated: foundation model adaptation building on MedSAM, SAM-Med2D, BiomedCLIP [82], BiomedGPT [83], RETFound [84] and Virchow [85]; federated learning across multiple centres [75,86]; multimodal integration of dermoscopy with clinical photographs, patient metadata and histopathology, leveraging the SLICE-3D benchmark [77]; self-supervised pretraining, especially valuable for under-represented skin tones where labelled data are scarce; continual learning under the FDA Predetermined Change Control Plan framework; and uncertainty quantification through deep ensembles, evidential deep learning and conformal prediction—an area where calibration alone is insufficient, as Section 3.8 illustrates.

### 4.7. Regulatory Considerations

The regulatory landscape for AI-enabled medical devices has matured rapidly. The FDA AI/ML-Enabled Medical Device List contained 1016 devices as of December 2024, with 510(k) clearance accounting for 94.6% of the 168 ML-enabled Class II devices cleared in 2024. The Predetermined Change Control Plan (PCCP) final guidance, issued on 4 December 2024, allows manufacturers to pre-specify and validate future modifications to AI-enabled devices through three components: a Description of Modifications, a Modification Protocol, and an Impact Assessment [87]. The Good Machine Learning Practice principles, jointly issued by the FDA, Health Canada, and the MHRA in 2021 [88], establish ten guiding principles. In the European Union, the Medical Device Regulation (EU 2017/745) classifies most AI-based diagnostic support tools under Rule 11 as Class IIa or IIb, and the EU AI Act (Regulation 2024/1689, in force August 2024) [89] establishes a high-risk classification with transparency, data governance, post-market surveillance, and human oversight obligations. The recent FUTURE-AI international consensus guideline [90] consolidates trustworthy and deployable AI requirements across six principles (Fairness, Universality, Traceability, Usability, Robustness, Explainability). Two of our findings bear on this framework directly. Calibration drift under cohort shift and the unreliability of single-parameter local correction (Section 3.8) are post-market surveillance problems rather than development problems, and they argue for monitoring plans that track calibration rather than accuracy alone. The explainability obligations of the AI Act are not discharged by producing a saliency map: based on the evidence of Section 3.7, a saliency map may be indistinguishable from a fixed circle by the metric most commonly used to validate it.

## 5. Conclusions

We evaluated a complete dermoscopic image analysis pipeline on HAM10000/ISIC 2018 under a protocol constructed so that each reported comparison isolates the factor it names, and validated it externally on BCN20000. Under lesion-level grouping, with input resolution and pretraining corpus held constant and the outer fold as the unit of analysis, six classification configurations spanning convolutional and transformer families are statistically indistinguishable (Friedman χ^2^ = 7.057, *p* = 0.216); no best architecture can be identified. The advantage of a promptable foundation model in segmentation is entirely an artefact of prompt provenance, disappearing when prompts are generated from the image alone. Three class activation methods score below a fixed, image-independent centred disc on the mask-IoU metric; so, this metric cannot support claims of lesion-focused attention. Cross-cohort transfer costs a mean of 25 balanced accuracy points, reverses the internal ranking, and is not reliably repaired by local recalibration. Temperature scaling remains worthwhile and cheap, reducing in-domain calibration error without affecting accuracy, but it does not transfer. The practical recommendations that follow are modest and inexpensive. Report a majority-class baseline alongside accuracy in imbalanced cohorts. Report a matched trivial baseline alongside any saliency–localisation score. State the prompting regime of any promptable model, and evaluate it end to end if a deployment claim is intended. Choose a unit of statistical analysis that is genuinely blocked, and do not count correlated repetitions as independent. State the sample underlying every column of an external-validation table. Deposit code, seeds, splits and weights under a DOI at submission rather than promising them on publication. None of these is a methodological innovation. Collectively, however, they account for the difference between the results we submitted and the results we report, and we would suggest that the leverage in dermoscopic deep learning currently lies less in architectural novelty than in the evaluation protocol that surrounds it.

## Figures and Tables

**Figure 1:**
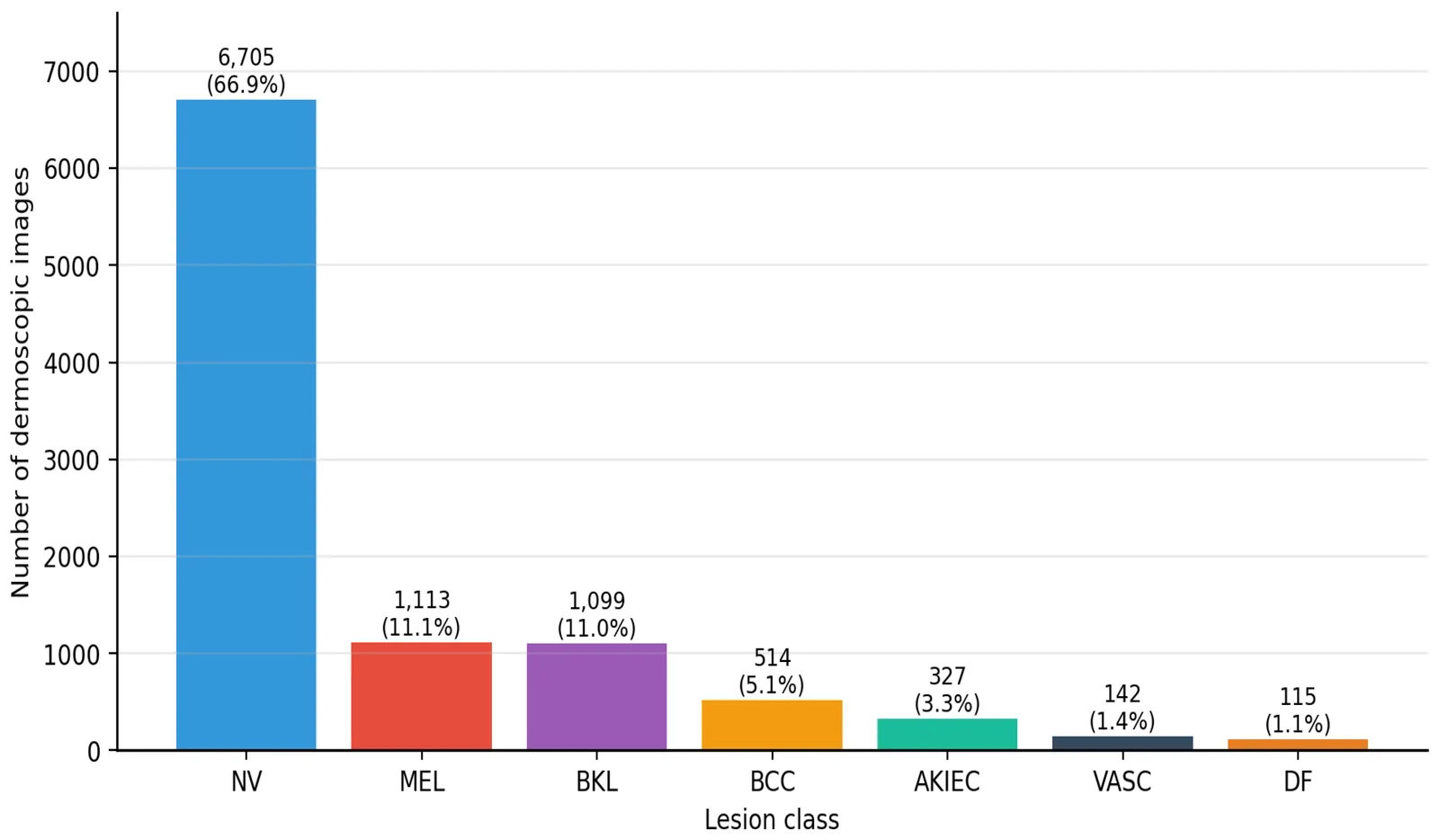
HAM10000 class distribution. Severe imbalance (NV ≈ 67% versus DF ≈ 1.15%) motivates focal loss training and a weighted random sampler.

**Figure 2:**
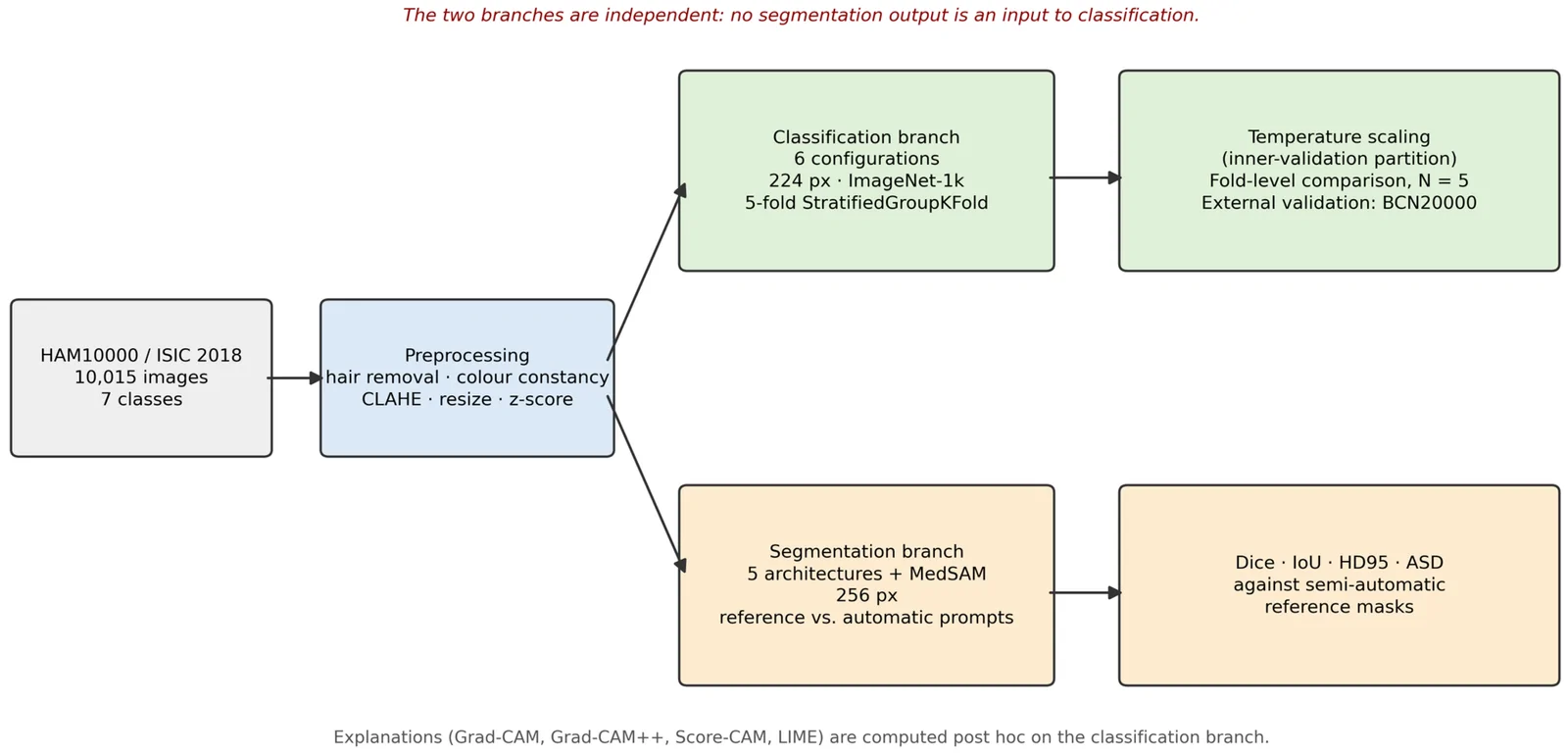
Study design. Preprocessing is shared, after which the classification and segmentation branches proceed independently: no segmentation output is an input to classification, and no result in this paper combines them. The classification branch comprises six configurations at a fixed 224 px input and fixed ImageNet-1k pretraining, evaluated under five-fold lesion-level StratifiedGroupKFold with temperature scaling fitted on an inner-validation partition. The segmentation branch comprises five architectures plus MedSAM, the latter evaluated separately under reference-derived and fully automatic box prompts. Explanations are computed post hoc on the classification branch. The submitted version showed an arrow from classification to segmentation, which had no computational meaning and has been removed.

**Figure 3:**
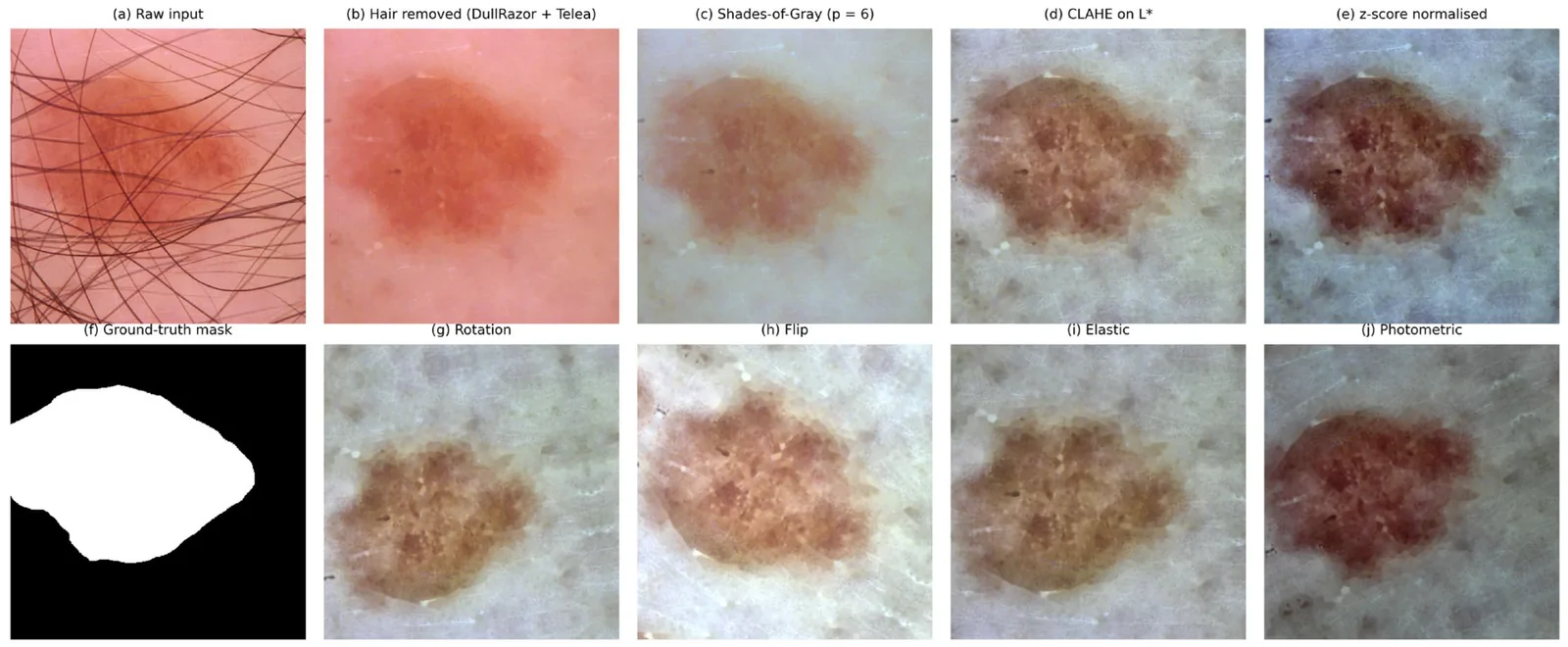
The preprocessing pipeline applied to a real dermoscopic image (ISIC_0024421), selected automatically as the most hair-dense candidate. Top row: (**a**) the raw input with dense hair artefacts; (**b**) DullRazor hair removal with Telea inpainting; (**c**) Shades-of-Gray colour constancy (*p* = 6); (**d**) CLAHE on the L* channel of CIE Lab*; (**e**) the z-score-normalised final input. Bottom row: (**f**) the reference lesion mask; (**g**–**j**) four independent draws from the augmentation policy—rotation, flip, elastic transform, and photometric jitter. Every panel is the actual output of the corresponding pipeline stage rather than a schematic. In panel (**b**), the hair pixel fraction is reduced by 98.7%, from 76.4% to 1.0% of the image.

**Figure 4:**
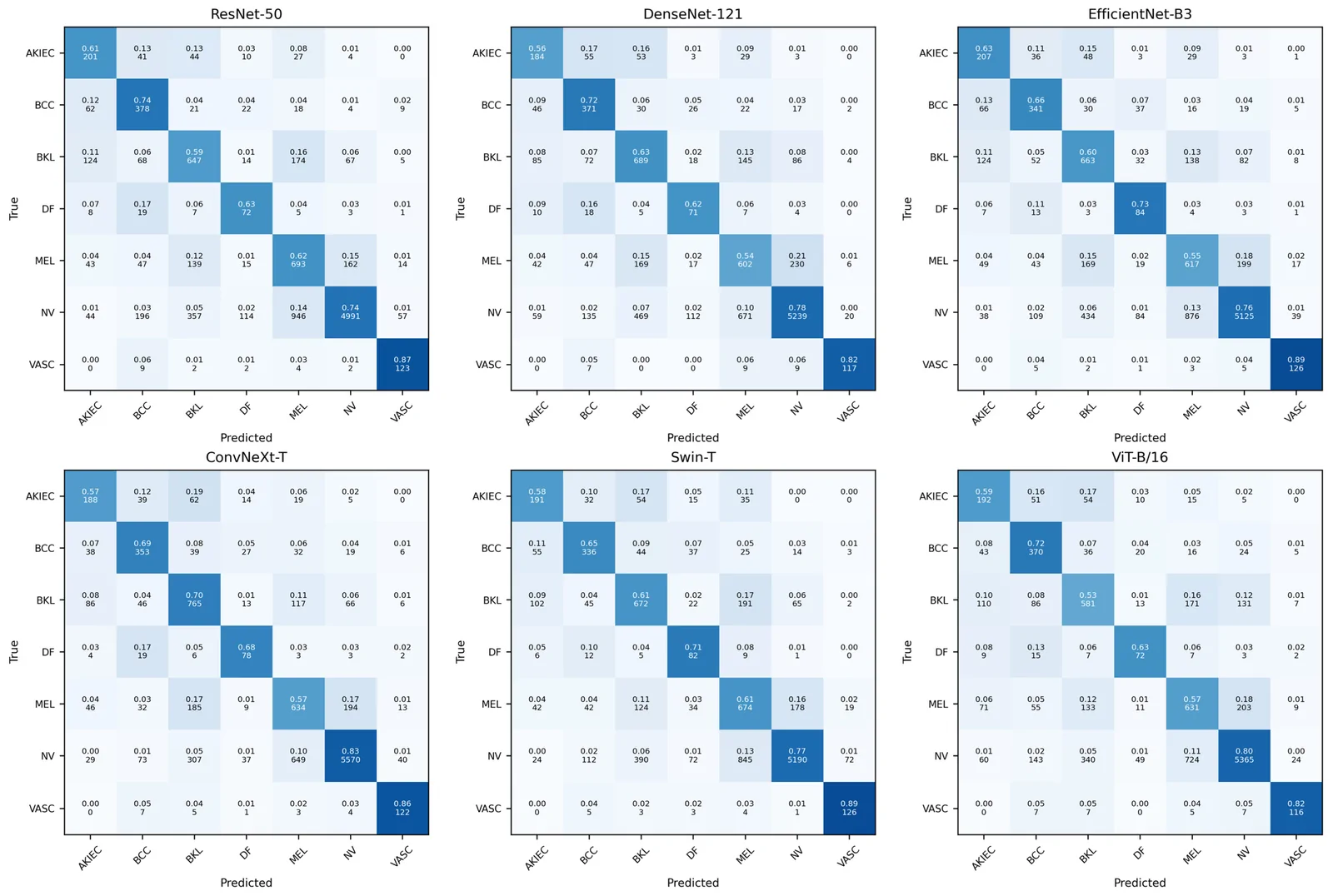
Row-normalised confusion matrices for all six configurations on the pooled held-out folds; each cell gives the row-normalised proportion above the raw count. No subset of models is singled out. The dominant residual error is melanoma classified as melanocytic nevus, affecting 16–21% of melanomas depending on configuration, and the rare classes (AKIEC, DF) remain the weakest despite focal loss and weighted sampling. Cell shading is proportional to the row-normalised proportion (darker blue indicates a higher proportion).

**Figure 5:**
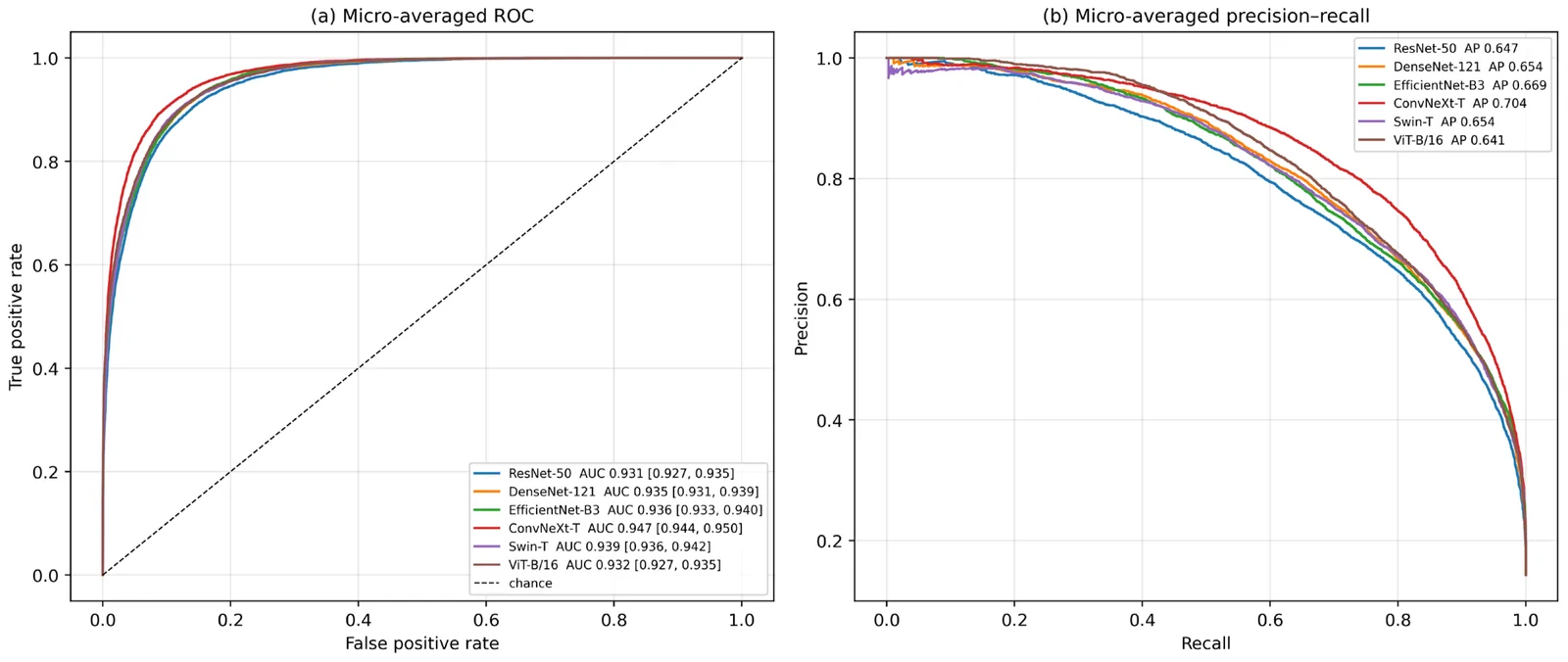
(**a**) Micro-averaged ROC and (**b**) micro-averaged precision–recall curves for the six configurations, computed on the pooled predictions of all five held-out folds after temperature scaling. Legend values are the macro-averaged one-versus-rest AUC with bootstrap 95% confidence intervals (B = 200) and the macro-averaged average precision. Because these are computed on pooled predictions, whereas Table 4 reports the mean of the per-fold values, the two differ slightly. The curves are closely superimposed, consistent with the equivalence result reported in Section 3.5.

**Figure 6:**
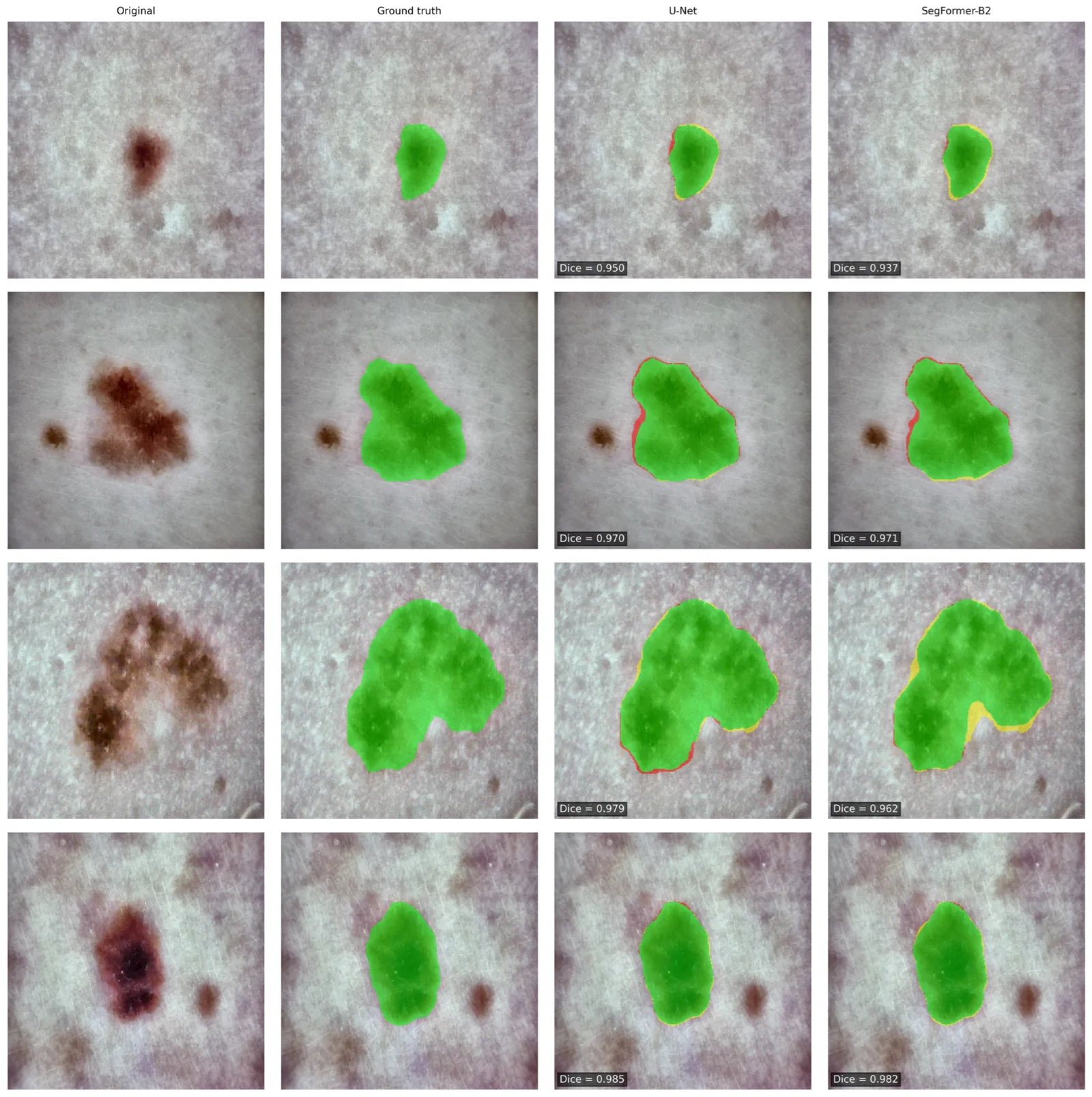
Qualitative segmentation results on four held-out cases sampled at the 20th, 40th, 60th and 80th percentiles of the Dice distribution, rather than from its upper tail. Columns: original image; reference mask overlay (green); U-Net prediction; SegFormer-B2 prediction. True positive = green, false positive = red, false negative = yellow. Per-image Dice is annotated on each panel and ranges from 0.937 to 0.985, consistent with the fold means in Table 5. Values are computed on dermoscopic images against the semi-automatic HAM10000 reference masks described in Section 2.1.

**Figure 7:**
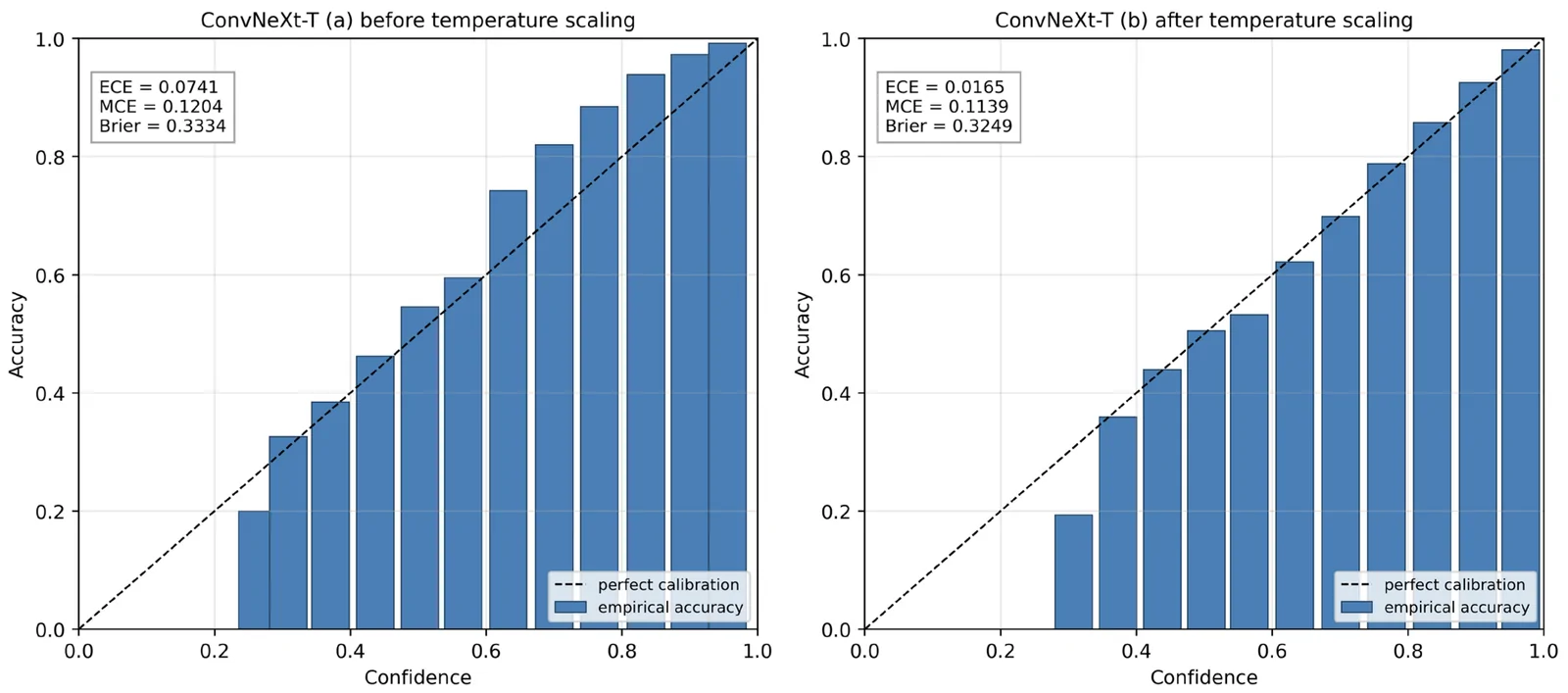
Reliability diagrams for ConvNeXt-T, the configuration with the highest mean fold balanced accuracy, on the pooled held-out folds: (**a**) before and (**b**) after temperature scaling. Bars give the empirical accuracy within each of fifteen equal-width confidence bins; the diagonal is perfect calibration. Temperature scaling reduces the expected calibration error from 0.0741 to 0.0165 without altering argmax accuracy. These values are computed on pooled predictions; Table 4 reports the mean of the per-fold values (0.0774 and 0.0274) and the two therefore differ slightly.

**Figure 8:**
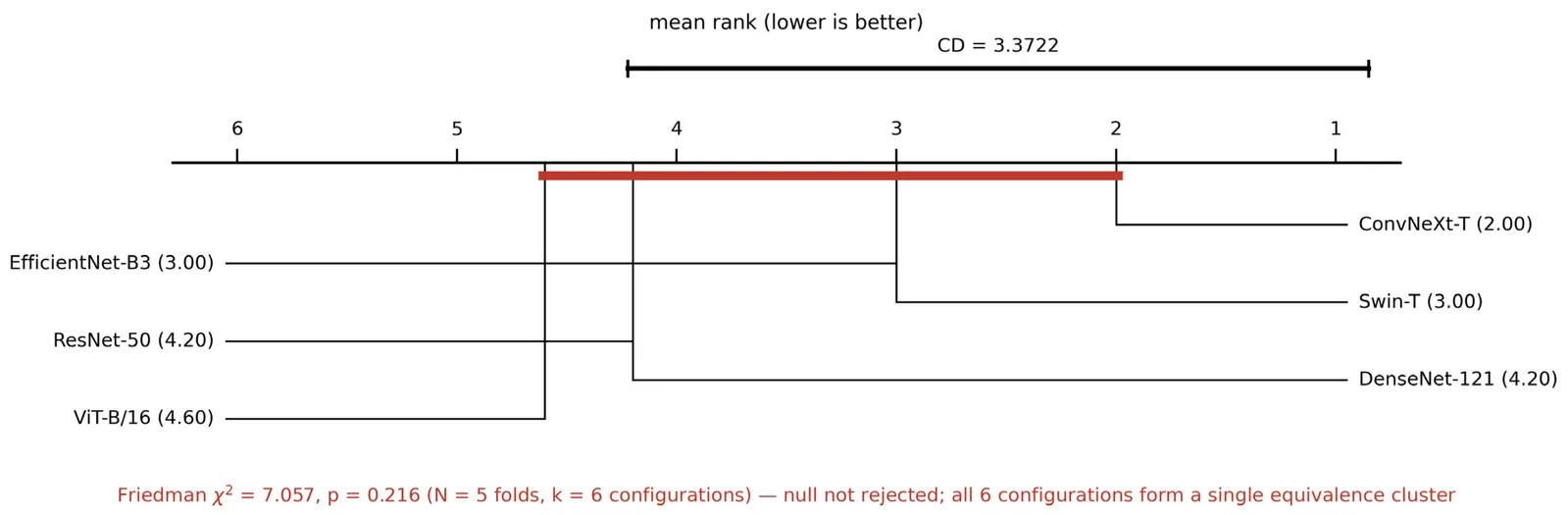
A critical-difference diagram for the six configurations across the five held-out folds (N = 5, k = 6). Mean ranks are computed on temperature-scaled balanced accuracy; lower is better. The Friedman omnibus test does not reject the null (χ^2^ = 7.057, *p* = 0.216), so no post hoc pairwise comparison is warranted and all six configurations are joined by a single equivalence bar (red line). The critical difference of 3.3722 exceeds the entire observed rank spread (2.00 to 4.60).

**Figure 9:**
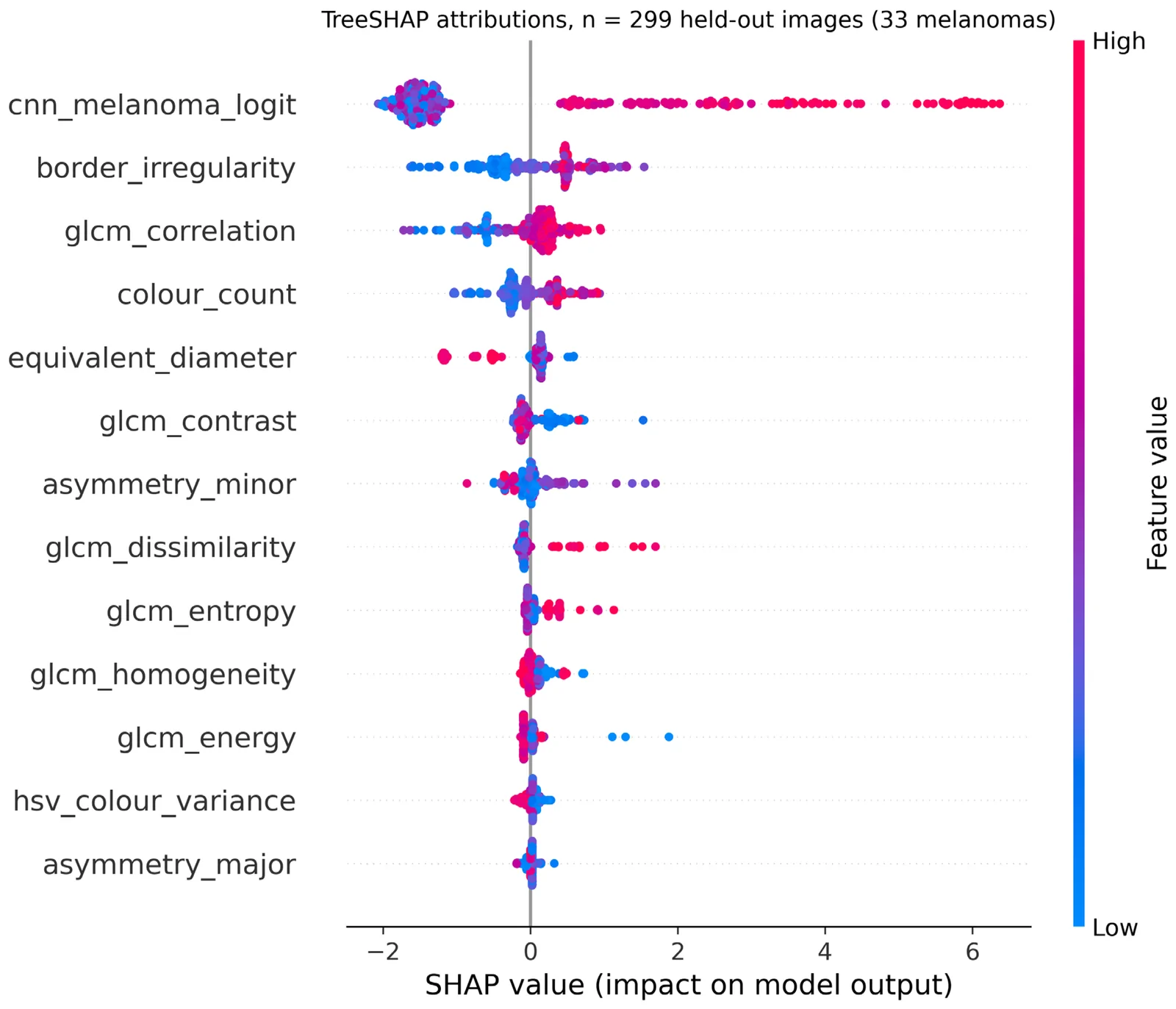
TreeSHAP attributions for the logistic model over the CNN melanoma logit plus twelve image-derived descriptors, on 299 held-out images of which 33 are melanomas. Each point is one image; the horizontal position is the SHAP value and the colour the feature value. The CNN logit dominates by roughly a factor of four over the next feature (mean absolute SHAP 1.99 versus 0.49 for border irregularity), and the twelve descriptors together change cross-validated discrimination by −0.0040 AUC. The features “blue-white veil” and “pigment network atypia” reported in the submitted version are not available as annotations in HAM10000 and have been removed; every feature shown is computed from the image and the predicted mask.

**Figure 10:**
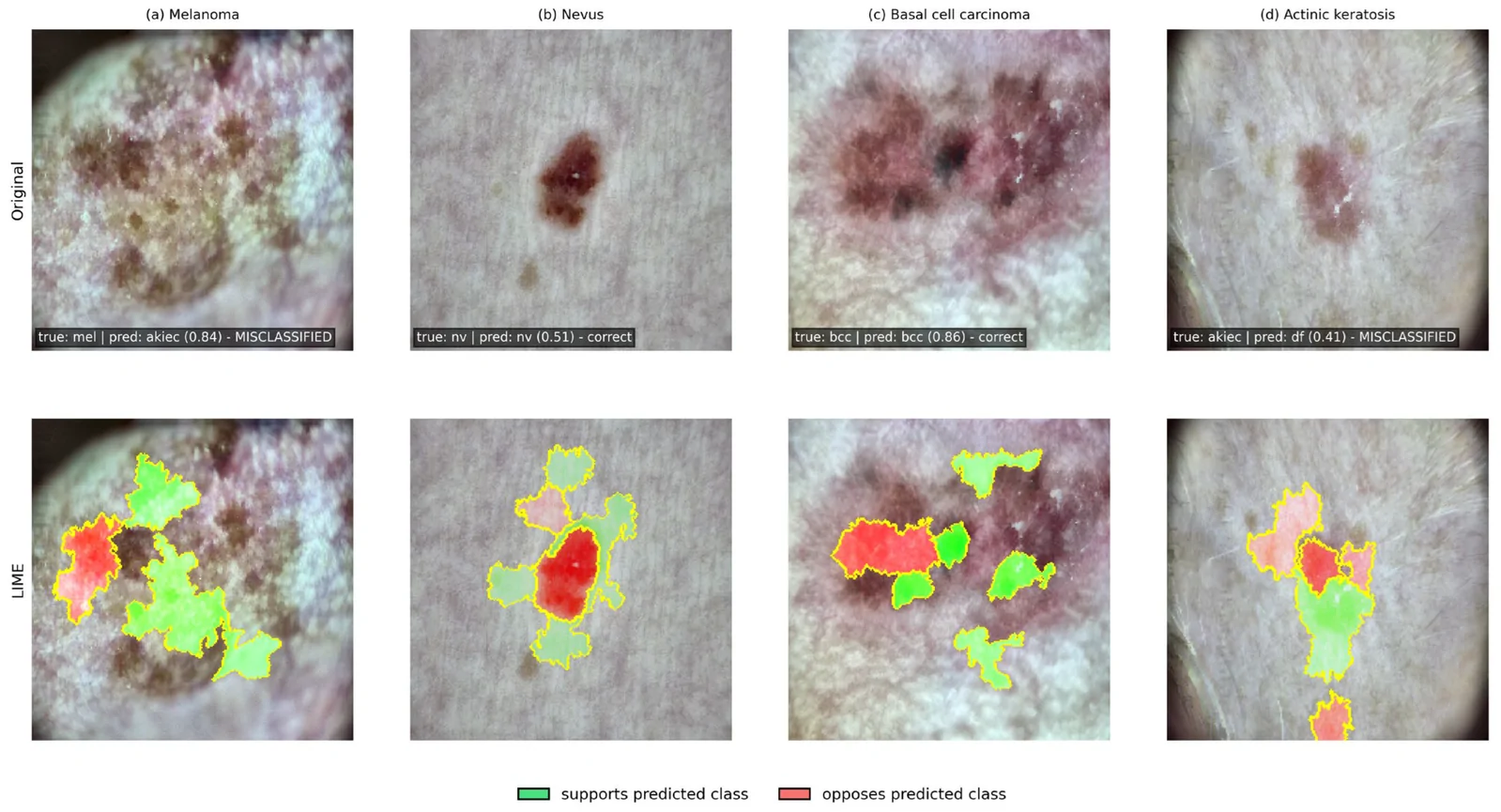
LIME explanations for four held-out dermoscopic cases, one per diagnostic class—(**a**) melanoma, (**b**) melanocytic nevus, (**c**) basal cell carcinoma, and (**d**) actinic keratosis. Panels were selected as the first case of each class in the held-out fold of the checkpoint used, without reference to whether the model classified them correctly. Top row: original images, annotated with the true class, the predicted class and its softmax probability. Bottom row: SLIC superpixel attributions, where green superpixels contribute positively to the predicted class and red negatively. Two of the four cases are misclassified, and in (**b**,**c**), the lesion core itself carries negative weight while the supporting superpixels lie in perilesional skin. Class abbreviations: mel, melanoma; nv, melanocytic nevus; bcc, basal cell carcinoma; akiec, actinic keratosis/intraepithelial carcinoma; df, dermatofibroma.

**Figure 11:**
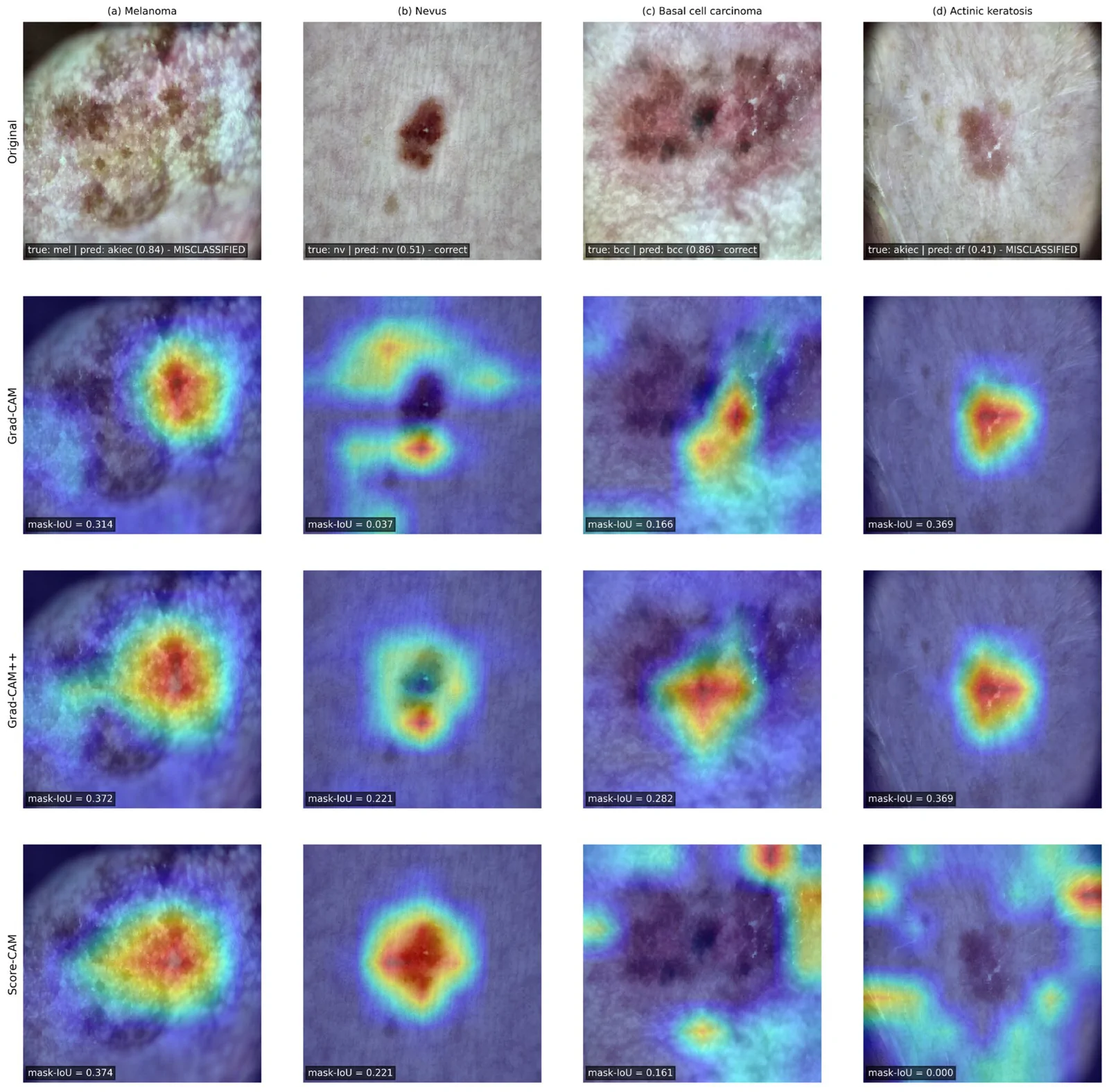
Grad-CAM, Grad-CAM++ and Score-CAM maps for the same four held-out cases and the same ConvNeXt-T checkpoint. Maps are computed for the predicted class, which in (**a**,**d**) is not the true class. Each panel reports the mask-IoU obtained by binarising the upper quintile of the saliency mass against the reference lesion mask, using the definition applied throughout Table 7. Panel means are 0.222 for Grad-CAM, 0.311 for Grad-CAM++ and 0.189 for Score-CAM; no panel reaches the fixed centred-disc baseline of 0.4745, and Score-CAM assigns its strongest activation to image corners outside the lesion in (**c**,**d**), yielding a mask-IoU of 0.000 in (**d**). The diamond-shaped contours in the Score-CAM row are an interpolation artefact of upsampling the 7 × 7 final-stage activation map to 224 × 224, not an anatomical structure. In the saliency maps, warmer colours (red) indicate higher activation and cooler colours (blue) lower activation. Columns: (**a**) melanoma; (**b**) melanocytic nevus; (**c**) basal cell carcinoma; (**d**) actinic keratosis.

**Table 1:** HAM10000/ISIC 2018 dataset composition: 10,015 images of 7470 unique lesions across seven diagnostic categories. Ground truth was established by histopathology, follow-up, expert consensus or in vivo confocal microscopy. Distributed under CC BY-NC 4.0.

Class	Code	Images	%	Ground-Truth Source
Melanocytic nevi	NV	6705	66.95	Histology/follow-up/consensus
Melanoma	MEL	1113	11.11	Histology
Benign keratosis-like lesions	BKL	1099	10.97	Histology/consensus
Basal cell carcinoma	BCC	514	5.13	Histology
Actinic keratoses and intraepithelial carcinoma	AKIEC	327	3.27	Histology
Vascular lesions	VASC	142	1.42	Consensus/confocal
Dermatofibroma	DF	115	1.15	Histology
Total	—	10,015	100.00	—

**Table 2:** Preprocessing pipeline. Steps 1–5 are deterministic and cached to disk once, so every configuration receives byte-identical inputs; step 6 is applied online during training only. The contribution of the individual steps was not ablated in this study (Section 4.4).

Step	Method	Parameters	Library
1	Hair removal	DullRazor: black-hat, cross SE 17 × 17, threshold 10; Telea inpainting r = 3	OpenCV 4.12
2	Colour constancy	Shades-of-Gray, Minkowski *p* = 6	NumPy
3	Contrast	CLAHE on L* of CIE L*a*b*, clip = 2.0, tiles 8 × 8	OpenCV 4.12
4	Resize	Bicubic to 224 × 224 (classification)/256 × 256 (segmentation)	OpenCV 4.12
5	Normalisation	Channel-wise z-score, ImageNet statistics	NumPy
6	Augmentation (train only)	H/V flip *p* = 0.5; rotation ±30°, scale ±10%, shift ±6.25% *p* = 0.7; elastic α = 120 *p* = 0.3; HSV shift (10, 15, 10) *p* = 0.5; brightness/contrast ±0.2 *p* = 0.5; coarse dropout 4 holes *p* = 0.2	Albumentations

**Table 3:** Training hyperparameters. Input resolution and pretraining corpus are held constant across all six configurations, so the comparison is not confounded by these factors. Early stopping patience was 15 epochs on inner-validation balanced accuracy.

Model	Optimiser	LR (init)	Schedule	Batch	Grad. Accum.	Epochs (Max)	Weight Decay	Input	Pretraining
ResNet-50	AdamW	1 × 10^−3^	OneCycle (cosine)	24	2	60	1 × 10^−4^	224 × 224	ImageNet-1k
DenseNet-121	AdamW	1 × 10^−3^	OneCycle (cosine)	24	2	60	1 × 10^−4^	224 × 224	ImageNet-1k
EfficientNet-B3	AdamW	1 × 10^−3^	OneCycle (cosine)	16	2	60	1 × 10^−4^	224 × 224	ImageNet-1k
ConvNeXt-T	AdamW	1 × 10^−4^	OneCycle (cosine)	16	2	60	0.05	224 × 224	ImageNet-1k
Swin-T	AdamW	1 × 10^−4^	OneCycle (cosine)	16	2	60	0.05	224 × 224	ImageNet-1k
ViT-B/16	AdamW	1 × 10^−4^	OneCycle (cosine)	8	2	60	0.05	224 × 224	ImageNet-1k

**Table 4:** Classification performance on the held-out outer folds, showing the mean over five folds with bootstrap BCa 95% confidence intervals. All metrics are computed after temperature scaling, where T was fitted on the inner-validation partition of the same fold and never on the test fold (Section 2.5 and Section 2.9). Selecting the top row on the basis of this same evaluation is upward-biased; the external estimate reported in Section 3.8 is the quantity that supports generalisation claims. The final row gives the majority-class baseline, which learns nothing and assigns every image to the most frequent class: accuracy in this cohort therefore has a floor near 0.67 and should not be read on its own, whereas balanced accuracy has a floor of 1/7 = 0.143. Bootstrap BCa 95% confidence intervals are shown for balanced accuracy, the primary metric; intervals for every metric and fold are in the deposited archive.

Model	Params (M)	Acc.	Bal. Acc. [95% CI]	Macro-F1	AUC	PR-AUC	MCC	Brier	ECE Pre-T	ECE Post-T	T
ConvNeXt-T	27.8	0.770	0.700 [0.674, 0.714]	0.640	0.950	0.724	0.607	0.325	0.0774	0.0274	0.757
EfficientNet-B3	10.7	0.715	0.691 [0.674, 0.706]	0.598	0.937	0.677	0.540	0.375	0.0509	0.0382	0.967
Swin-T	27.5	0.726	0.690 [0.674, 0.704]	0.594	0.944	0.681	0.557	0.371	0.0519	0.0279	0.848
ResNet-50	23.5	0.709	0.686 [0.653, 0.721]	0.585	0.933	0.655	0.542	0.393	0.0487	0.0273	0.812
DenseNet-121	7.0	0.726	0.668 [0.612, 0.699]	0.598	0.937	0.664	0.548	0.370	0.0405	0.0279	0.855
ViT-B/16	85.8	0.732	0.664 [0.632, 0.693]	0.596	0.936	0.658	0.548	0.360	0.0596	0.0354	0.874
Majority-class baseline (always NV)	—	0.669	0.143	0.115	0.500	0.669	0.000	0.331	—	—	—

**Table 5:** Segmentation performance on the held-out fold, evaluated against the semi-automatic HAM10000 lesion masks of Tschandl et al. (network-generated and subsequently corrected by a dermatologist; not expert-drawn). MedSAM is reported under two prompting regimes: reference-derived boxes give an upper bound and are not comparable with the unprompted networks, whereas fully automatic boxes use no reference information and constitute the only like-for-like comparison. HD95 = 95th-percentile Hausdorff distance; ASD = average surface distance; Sens. = sensitivity; Spec. = specificity.

Model	Params (M)	Dice	IoU	HD95 (px)	ASD (px)	Sens.	Spec.
MedSAM—reference-derived box prompts (upper bound, not comparable)	93.7	0.9547	0.9147	6.38	1.40	0.9617	0.9758
SegFormer-B2	27.3	0.9416	0.8971	8.56	1.98	0.9393	0.9777
U-Net++	26.1	0.9406	0.8955	8.86	2.01	0.9454	0.9720
U-Net	24.4	0.9402	0.8947	8.80	2.01	0.9451	0.9743
Attention U-Net	24.6	0.9401	0.8952	9.14	2.09	0.9493	0.9739
DeepLabV3+	26.7	0.9332	0.8848	9.94	2.37	0.9342	0.9766
MedSAM—fully automatic box prompts (comparable)	93.7	0.7729	0.7019	35.29	14.15	0.8915	0.8768

**Table 6:** Multi-configuration comparison. The unit of analysis is the outer fold (N = 5), the only genuinely blocked unit that this design provides; the submitted version treated 5 folds × 3 seeds as 15 independent observations, which they are not, since folds share three-quarters of their training data and seeds within a fold share a partition. The omnibus test does not reject the null, so no post hoc pairwise comparison is warranted and the equivalence cluster spans all six configurations.

Analysis	Blocks (N)	Friedman χ^2^	*p*	Critical Difference	Equivalence Cluster
Fold-level (one seed per fold, k = 6)	5	7.057	0.216	3.3722	ConvNeXt-T, EfficientNet-B3, Swin-T, DenseNet-121, ResNet-50, ViT-B/16

**Table 7:** Saliency localisation. Mask-IoU binarises the upper quintile of the saliency mass and intersects it with the reference lesion mask. Because dermoscopic lesions are large and approximately centred, the metric has a high floor, so absolute values are uninterpretable without a matched trivial baseline; a fixed, image-independent centred disc scores 0.4745 on the same 300 images (0.4898 across all 10,015 masks). All three class activation methods fall below that disc. The inference in the submitted version that these values demonstrate lesion-focused attention is withdrawn.

Method	Mask-IoU	Margin over Baseline
Centred-disc baseline (fixed, image-independent)	0.4745	—
Grad-CAM++	0.3512	−0.1233
Score-CAM	0.3351	−0.1393
Grad-CAM	0.2531	−0.2213

**Table 8:** External validation on the BCN20000 test split (*n* = 15,172 images). Internal Bal. Acc. is the mean over the five held-out development folds and Drop the difference. Samples underlying each column: accuracy, balanced accuracy, macro-F1, AUC, MCC, Brier and ECE (no adaptation) are computed on the test split using the HAM10000-fitted temperature with no cohort-specific adaptation; ECE (after local re-fit) is computed on those same images after re-fitting the temperature on a disjointed 961-image calibration split that is excluded from every other column. The two ECE columns are therefore not directly comparable with each other. The evaluation covers six of the seven HAM10000 classes: vascular lesions are absent from the labelled BCN20000 release, as are squamous cell carcinoma and out-of-distribution lesions, so these figures do not describe the full BCN20000 spectrum. The final row gives the majority-class baseline for this cohort.

Model	Internal Bal. Acc.	Bal. Acc.	Drop	Acc.	Macro-F1	AUC	MCC	Brier	ECE (No Adaptation)	ECE (After Local Re-Fit)
Swin-T	0.690	0.476	−0.214	0.554	0.432	0.839	0.419	0.586	0.0358	0.0374
ConvNeXt-T	0.700	0.451	−0.249	0.550	0.430	0.840	0.412	0.605	0.0546	0.0459
ResNet-50	0.686	0.435	−0.251	0.541	0.436	0.829	0.403	0.598	0.0286	0.0371
EfficientNet-B3	0.691	0.416	−0.275	0.542	0.402	0.815	0.388	0.592	0.0407	0.0434
ViT-B/16	0.664	0.410	−0.254	0.524	0.405	0.818	0.379	0.620	0.0383	0.0407
DenseNet-121	0.668	0.401	−0.267	0.518	0.398	0.802	0.363	0.627	0.0576	0.0394
Majority-class baseline (always NV)	—	0.167	—	0.353	—	0.500	0.000	—	—	—

## Data Availability

The HAM10000/ISIC 2018 dataset is publicly available at https://challenge.isic-archive.com/data/#2018 (accessed on 21 June 2026) and in the Harvard Dataverse (https://doi.org/10.7910/DVN/DBW86T, accessed on 21 June 2026). BCN20000 is available through the ISIC Archive under CC-BY-NC 4.0. The complete analysis code, the random seeds, the lesion-level fold assignment files, the trained weights for every reported configuration, the per-fold raw metrics, logits and fitted temperatures, and the scripts that generate every table and figure in this manuscript are archived and citable at https://doi.org/10.5281/zenodo.22654523. The archive is public at the date of submission. Image data are not redistributed, since the source cohorts carry their own licences; the archive documents how to obtain each, and the preprocessing is deterministic and cached so that the exact model inputs can be regenerated.

## Supplementary Materials

The following supporting information can be downloaded at https://www.mdpi.com/article/10.3390/jcm15197422/s1, Table S1: TRIPOD + AI reporting checklist [91].

## Abbreviations

The following abbreviations are used in this manuscript:

ABCDE	Asymmetry, Border, Colour, Diameter, Evolution (dermatological rule)
ACE	Adaptive Calibration Error
AI	Artificial intelligence
AKIEC	Actinic keratoses and intraepithelial carcinoma
AOPC	Area Over the Perturbation Curve
ASD	Average Surface Distance
AUC	Area Under the Curve
BCa	Bias-corrected and accelerated (bootstrap)
BCC	Basal cell carcinoma
BCE	Binary cross-entropy
BCN20000	Barcelona dermoscopic dataset (Hospital Clínic Barcelona
BKL	Benign keratosis-like lesion
BS	Brier score
CC-BY-NC	Creative Commons Attribution—NonCommercial
CD	Critical difference
CI	Confidence interval
CIE	International Commission on Illumination (CIE L*a*b* colour space)
CLAHE	Contrast Limited Adaptive Histogram Equalization
CNN	Convolutional neural network
CUDA	Compute Unified Device Architecture
cuDNN	CUDA Deep Neural Network library
DeiT	Data-efficient image transformer
DF	Dermatofibroma
Df	Degrees of freedom
DSC	Dice similarity coefficient
ECE	Expected calibration error
EU	European Union
FDA	(US) Food and Drug Administration
FDR	False discovery rate
FN, FP	False negative, false positive
FWER	Familywise Error Rate
GLCM	Gray-Level Co-occurrence Matrix
GMLP	Good Machine Learning Practice
GPU	Graphics Processing Unit
Grad-CAM	Gradient-weighted Class Activation Mapping
HAM10000	Human Against Machine with 10,000 training images
HD95	95th-percentile Hausdorff Distance
HSV	Hue, Saturation, Value
IoU	Intersection over Union
IRB	Institutional Review Board
ISIC	International Skin Imaging Collaboration
L-BFGS	Limited-memory Broyden–Fletcher–Goldfarb–Shanno algorithm
LIME	Local Interpretable Model-agnostic Explanations
MAE	Masked Autoencoder
MCC	Matthews Correlation Coefficient
MCE	Maximum Calibration Error
MDR	Medical Device Regulation
MedSAM	Medical Segment Anything Model
MEL	Melanoma
MHRA	Medicines and Healthcare products Regulatory Agency
ML	Machine learning
MONAI	Medical Open Network for Artificial Intelligence
nnU-Net	Self-configuring no-new-U-Net
NPV	Negative predictive value
NV	Melanocytic nevi
PCCP	Predetermined Change Control Plan
PH^2^	Dermoscopic image database (Hospital Pedro Hispano)
PR-AUC	Precision–Recall Area Under the Curve
RGB	Red, Green, Blue
ROC	Receiver Operating Characteristic
SAM	Segment Anything Model
SCC	Squamous cell carcinoma
Score-CAM	Score-weighted Class Activation Mapping
SGD	Stochastic Gradient Descent
SHAP	SHapley Additive exPlanations
SLIC	Simple Linear Iterative Clustering
TN	True negative
TP	True positive
TRIPOD + AI	Transparent Reporting of a multivariable prediction model for Individual Prognosis Or Diagnosis—AI extension
VASC	Vascular lesion
ViT	Vision Transformer
XAI	Explainable artificial intelligence
