# Supplementary material for: A Reproducible and Explainable Deep Learning Framework for Dermoscopic Image Analysis in Skin Cancer
Source: J Clin Med. 2026 Sep 24;15(19):7422. doi: 10.3390/jcm15197422 (PMC13666333; doi:10.3390/jcm15197422)
Supplement: Supplementary file 1 [file jcm-15-07422-s001.zip › jcm-4511343-supplementary.pdf]

Table S1. TRIPOD+AI reporting checklist

Completed using the official TRIPOD+AI checklist (version 11 January 2024) obtained from tripod-statement.org. Source: Collins GS, Moons KGM, Dhiman P, et al. TRIPOD+AI statement: updated guidance for reporting clinical prediction models that use regression or machine learning methods. BMJ 2024;385:e078378. doi:10.1136/bmj-2023-078378. Item numbering, sub-item structure and checklist wording are reproduced unaltered; no item has been renumbered, merged, abridged or omitted, and no text from the superseded TRIPOD 2015 checklist is used. D = item relevant only to model development; E = item relevant only to model evaluation; D;E = applicable to both. Items the study does not meet are marked as such rather than omitted.

Section / Topic	Item	D;E	Checklist item	Where reported in the revised manuscript
**TITLE**				
**Title**	1	D;E	Identify the study as developing or evaluating the performance of a multivariable prediction model, the target population, and the outcome to be predicted	Title, p. 1. The title identifies the study as developing and externally evaluating deep learning models for dermoscopic skin lesion analysis. The target population (patients with pigmented skin lesions imaged at specialist dermatology centres) and the outcome (seven-class lesion diagnosis) are stated in the Abstract rather than the title.
**ABSTRACT**				
**Abstract**	2	D;E	See TRIPOD+AI for Abstracts checklist	Abstract, p. 1. Structured abstract reporting objectives, data sources, design, sample size, model types, analytical methods, performance, calibration, external validation and conclusions.
**INTRODUCTION**				
**Background**	3a	D;E	Explain the healthcare context (including whether diagnostic or prognostic) and rationale for developing or evaluating the prediction model, including references to existing models	Section 1, paragraphs 1–5. Diagnostic context. Existing models are reviewed (DSCC_Net, SkinSage XAI, SkinFormer, SapFormer, DermViT, Swin-UNet, TransUNet, BiADATU-Net and others) and the rationale is methodological rather than architectural: to measure how much of the reported performance of such pipelines survives an evaluation protocol designed to remove optimism.
	3b	D;E	Describe the target population and the intended purpose of the prediction model in the context of the care pathway, including its intended users (e.g., healthcare professionals, patients, public)	Sections 1 and 4.5. Target population: patients presenting with pigmented skin lesions already selected for dermoscopic imaging at specialist centres. Intended purpose: the revised manuscript withdraws the triage proposal of the submitted version and states that the evidence supports, at most, a ranking or prioritisation aid under local threshold calibration, not an autonomous classifier. Intended users would be clinicians; no deployment claim is made.
	3c	D;E	Describe any known health inequalities between sociodemographic groups	Section 1, paragraph 2 (unequal access to dermatological assessment among the United States population aged 65 and over, per the cited Medicare-participation analysis) and Section 4.4 (under-representation of Fitzpatrick skin types III–VI in both cohorts, with reference to documented performance disparities across skin tones).
**Objectives**	4	D;E	Specify the study objectives, including whether the study describes the development or validation of a prediction model (or both)	Section 1, final two paragraphs. The study both develops (five-fold cross-validation on HAM10000 / ISIC 2018) and externally evaluates (BCN20000) the models. Four explicitly methodological objectives are stated.
**METHODS**				
**Data**	5a	D;E	Describe the sources of data separately for the development and evaluation datasets (e.g., randomised trial, cohort, routine care or registry data), the rationale for using these data, and representativeness of the data	Section 2.1. Development: HAM10000 / ISIC 2018, an archival multi-source dermoscopic collection. Evaluation: BCN20000, an independent archival collection from a different centre, device and population. Rationale: both are public, permitting full reproducibility. Representativeness is addressed in Section 4.4: both cohorts are European, specialist-centre and dermoscopic, and neither is a consecutive clinical series. No synthetic data were used.
	5b	D;E	Specify the dates of the collected participant data, including start and end of participant accrual; and, if applicable, end of follow-up	Section 2.1. HAM10000 images were accrued over a 20-year period at the two contributing centres; BCN20000 images between 2010 and 2016 at Hospital Clínic de Barcelona. Both are archival collections; no participant follow-up applies, since this is a diagnostic rather than a prognostic model.
**Participants**	6a	D;E	Specify key elements of the study setting (e.g., primary care, secondary care, general population) including the number and location of centres	Section 2.1. Secondary/tertiary specialist dermatology care. Development data from two centres (Medical University of Vienna, Austria; University of Queensland, Australia); evaluation data from one centre (Hospital Clínic de Barcelona, Spain).
	6b	D;E	Describe the eligibility criteria for study participants	Section 2.1. No eligibility criteria were applied by us: all images in the public releases were used. Eligibility was therefore determined by the source cohorts, in which lesions had already been selected for dermoscopic imaging by specialists. Section 4.4 states the consequence — prevalence and case mix differ from an unselected population and positive predictive value would fall accordingly.
	6c	D;E	Give details of any treatments received, and how they were handled during model development or evaluation, if relevant	Not applicable. No treatment information is recorded in either cohort and no treatment was modelled.
**Data preparation**	7	D;E	Describe any data pre-processing and quality checking, including whether this was similar across relevant sociodemographic groups	Section 2.3 and Table 2; Figure 3 shows every stage on a real image. Five deterministic stages are computed once and cached, so all configurations receive byte-identical inputs. All preprocessing code is in the deposited archive. Whether preprocessing behaved similarly across sociodemographic groups could not be assessed:

				Fitzpatrick phototype is not recorded in either cohort (Section 4.4, item 14).
**Outcome**	8a	D;E	Clearly define the outcome that is being predicted and the time horizon, including how and when assessed, the rationale for choosing this outcome, and whether the method of outcome assessment is consistent across sociodemographic groups	Section 2.1 and Table 1. Outcome: the seven-class HAM10000 diagnostic label (reference standard established by histopathology, follow-up, expert consensus or in-vivo confocal microscopy). Diagnostic model; no time horizon applies. Consistency of outcome assessment across sociodemographic groups is not reported in the source cohorts and could not be verified here.
	8b	D;E	If outcome assessment requires subjective interpretation, describe the qualifications and demographic characteristics of the outcome assessors	Not reported, and outside our control. Outcome labels were assigned by the source cohorts before our analysis; the qualifications and demographic characteristics of those assessors are not published in the dataset descriptors. Section 4.4 notes that no clinician involved in the present study read any image.
	8c	D;E	Report any actions to blind assessment of the outcome to be predicted	Not applicable to the present analysis. Labels were fixed in the public releases before any model was trained, so outcome assessment could not have been influenced by the predictors or by the modelling.
**Predictors**	9a	D	Describe the choice of initial predictors (e.g., literature, previous models, all available predictors) and any pre-selection of predictors before model building	Sections 2.3 and 2.6. The sole input to the diagnostic model is the preprocessed dermoscopic image; no tabular predictors were used and no predictor pre-selection was performed. Patient metadata (age, sex, site) were deliberately not fused (Section 4.4). The twelve radiomic descriptors of Section 2.10 belong to a post-hoc interpretability analysis of the model output, not to the diagnostic model.
	9b	D;E	Clearly define all predictors, including how and when they were measured (and any actions to blind assessment of predictors for the outcome and other predictors)	Sections 2.3.1–2.3.4. The predictor is a 224 × 224 RGB image obtained by a fixed deterministic chain (hair removal, Shades-of-Gray colour constancy, CLAHE on L*, bicubic resize, ImageNet z-score). Measurement precedes labelling in both cohorts. Section 2.10 defines the twelve post-hoc descriptors and states that all are computed automatically from the image and the predicted mask.
	9c	D;E	If predictor measurement requires subjective interpretation, describe the qualifications and demographic characteristics of the predictor assessors	Not applicable. No predictor requires subjective interpretation: the input is the image itself and every derived quantity is computed programmatically. The submitted version listed “blue-white veil” and “pigment network atypia”, which would have required expert reading; these are not available as annotations in HAM10000 and have been removed (Section 2.10).
**Sample size**	10	D;E	Explain how the study size was arrived at (separately for development and evaluation), and justify that the study size was sufficient to answer the research question. Include details of any sample size calculation	Sections 2.1 and 2.5. No formal sample size calculation was performed; all available images were used (development 10,015; evaluation 15,172 plus a disjoint 961-image calibration split). Section 3.5 and Section 4.4 state the consequence for the primary comparison: with five folds as the unit of analysis the study is under-powered to separate configurations, and the reported failure to reject is explicitly not a demonstration of equality.
**Missing data**	11	D;E	Describe how missing data were handled. Provide reasons for omitting any data	Sections 2.1 and 3.7. No image was excluded for missingness in the classification or segmentation analyses. In the post-hoc radiomic analysis one of 300 images was lost to mask failure, leaving 299; this is stated in Section 3.7 and in the Figure 10 caption.
**Analytical methods**	12a	D	Describe how the data were used (e.g., for development and evaluation of model performance) in the analysis, including whether the data were partitioned, considering any sample size requirements	Section 2.5. Five-fold StratifiedGroupKFold at the lesion level; within each outer fold, the four training folds are further split 90/10 at the lesion level into training and inner-validation. Grouping by lesion_id prevents duplicate-lesion leakage across partitions. The fold assignment files are in the deposited archive, so the partitions can be reconstructed image by image.
	12b	D	Depending on the type of model, describe how predictors were handled in the analyses (functional form, rescaling, transformation, or any standardisation)	Section 2.3.4. Bicubic resize to a fixed 224 × 224 for classification and 256 × 256 for segmentation, followed by per-channel z-score standardisation with ImageNet statistics. Resolution is held constant across all configurations so that architecture is not confounded with input size (Section 2.6).
	12c	D	Specify the type of model, rationale, all model-building steps, including any hyperparameter tuning, and method for internal validation	Sections 2.6 and 2.7, Table 3. Six classification configurations (ResNet-50, DenseNet-121, EfficientNet-B3, ConvNeXt-T, Swin-T, ViT-B/16), all at fixed resolution and fixed ImageNet-1k pretraining; five segmentation architectures plus MedSAM under two prompting regimes. AdamW with a OneCycle schedule, fixed hyperparameters per family, early stopping on inner-validation balanced accuracy. Internal validation: five-fold lesion-level cross-validation with one seed per fold. No architecture search or hyperparameter sweep was performed; the model set was fixed before results were examined.
	12d	D;E	Describe if and how any heterogeneity in estimates of model parameter values and model performance was handled and quantified across clusters (e.g., hospitals, countries). See TRIPOD-Cluster for additional considerations	Section 2.11 and Section 3.8. No cluster-level random effects were modelled. Heterogeneity is quantified in two ways: between folds, which is the blocking unit of the Friedman analysis (N = 5), and between cohorts, reported as the 25-point mean drop in balanced accuracy and the reversal of the internal ranking on BCN20000.
	12e	D;E	Specify all measures and plots used (and their rationale) to evaluate model performance (e.g., discrimination, calibration, clinical utility) and, if relevant, to compare multiple models	Sections 2.8, 2.9 and 2.11. Discrimination (accuracy, balanced accuracy, macro-F1, MCC, ROC-AUC, PR-AUC), calibration (ECE, MCE, ACE, Brier, reliability diagrams), segmentation (Dice, IoU, HD95, ASD). A majority-class baseline is reported alongside accuracy because 66.95% of the development cohort belongs to one class. Model comparison: Friedman at the level of the outer fold with Nemenyi post-hoc only if the omnibus test rejects.
	12f	E	Describe any model updating (e.g., recalibration) arising from the model evaluation, either overall or for particular sociodemographic	Sections 2.5 and 2.9. A disjoint 961-image BCN20000 calibration split, excluded from every performance metric, was used to re-fit the temperature. No other updating was performed and no sociodemographic-specific

			groups or settings	recalibration was possible (item 14). Results in Section 3.8 and Table 8.
	12g	E	For model evaluation, describe how the model predictions were calculated (e.g., formula, code, object, application programming interface)	Section 2.2 and Data Availability Statement. The models have no closed-form equation. Predictions on the evaluation cohort were computed by loading the deposited per-fold weights and averaging the softmax outputs across the five fold checkpoints after temperature scaling. Weights, inference code, fitted temperatures and the raw test logits are archived at https://doi.org/10.5281/zenodo.22654523, so any third party can recompute the reported predictions.
**Class imbalance**	13	D;E	If class imbalance methods were used, state why and how this was done, and any subsequent methods to recalibrate the model or the model predictions	Section 2.4. The cohort is severely imbalanced (66.95% NV versus 1.15% DF). A weighted random sampler with inverse-frequency weights and a class-balanced focal loss ($\gamma = 2$) were used. Because imbalance correction distorts calibration, post-hoc temperature scaling was applied (Section 2.9) and calibration is reported before and after in Table 4.
**Fairness**	14	D;E	Describe any approaches that were used to address model fairness and their rationale	Section 4.4. No fairness analysis was performed and none was possible: neither cohort records Fitzpatrick phototype, ethnicity or other sociodemographic attributes in a form permitting stratified evaluation. This is stated as a limitation, with reference to documented performance disparities across skin tones, and the manuscript makes no claim of equitable performance.
**Model output**	15	D	Specify the output of the prediction model (e.g., probabilities, classification). Provide details and rationale for any classification and how the thresholds were identified	Sections 2.8.1 and 2.9. The output is a temperature-scaled seven-class probability vector; the reported classification is the argmax, with no tuned decision threshold and no risk grouping. The four-way triage stratification proposed in the submitted version has been withdrawn (Section 4.5), so no clinical thresholds are defined.
**Development versus evaluation**	16	D;E	Identify any differences between the development and evaluation data in healthcare setting, eligibility criteria, outcome, and predictors	Sections 2.1 and 3.8, Table 8 caption. Differences: different centre, country, dermatoscope and acquisition protocol; different class prevalence; and six of the seven classes only, since vascular lesions are absent from the labelled BCN20000 release, as are squamous cell carcinoma and out-of-distribution lesions. Outcome definition and the image predictor are otherwise comparable. Section 4.2 attributes the collapse in threshold-dependent performance to the prevalence shift.
**Ethical approval**	17	D;E	Name the institutional research board or ethics committee that approved the study and describe the participant-informed consent or the ethics committee waiver of informed consent	Section 2.1 (Ethical statement) and the Institutional Review Board Statement. Original data collection was approved by the institutional review board of the Medical University of Vienna (protocol 1804/2017) and by the University of Queensland. No separate approval was required for this secondary analysis of fully anonymised, publicly released data, and no individual consent was obtainable or required.
**OPEN SCIENCE**				
**Funding**	18a	D;E	Give the source of funding and the role of the funders for the present study	Funding statement in the back matter.
**Conflicts of interest**	18b	D;E	Declare any conflicts of interest and financial disclosures for all authors	Conflicts of Interest statement in the back matter.
**Protocol**	18c	D;E	Indicate where the study protocol can be accessed or state that a protocol was not prepared	No study protocol was prepared. This is a retrospective secondary analysis of public datasets and no protocol was written or registered in advance.
**Registration**	18d	D;E	Provide registration information for the study, including register name and registration number, or state that the study was not registered	The study was not registered.
**Data sharing**	18e	D;E	Provide details of the availability of the study data	Data Availability Statement. HAM10000 / ISIC 2018 is available from the ISIC Archive and Harvard Dataverse (doi:10.7910/DVN/DBW86T) under CC BY-NC 4.0; BCN20000 from the ISIC Archive under CC BY-NC 4.0. Image data are not redistributed because the source licences do not permit it; the deposited archive at https://doi.org/10.5281/zenodo.22654523 documents how to obtain each cohort and contains the lesion-level fold assignment files, so the exact partitions can be reconstructed.
**Code sharing**	18f	D;E	Provide details of the availability of the analytical code	Data Availability Statement. Complete analysis code (data preparation, preprocessing, training, evaluation, explainability, statistics, table and figure generation), the random seeds, the fold assignments, the trained weights for every reported configuration, the per-fold metrics, the raw test logits and the fitted temperatures are archived at https://doi.org/10.5281/zenodo.22654523 under the MIT licence, with package versions pinned in the deposited requirements file. The archive is public as of the date of submission and is not conditional on acceptance.
**PATIENT & PUBLIC INVOLVEMENT**				
**Patient & Public Involvement**	19	D;E	Provide details of any patient and public involvement during the design, conduct, reporting, interpretation, or dissemination of the study or state no involvement	No patients or members of the public were involved in the design, conduct, reporting, interpretation or dissemination of this study.
**RESULTS**				
**Participants**	20a	D;E	Describe the flow of participants through the study, including the number of participants with and without the outcome and, if	Section 2.1, Table 1 and Figure 1 for the development cohort (all 10,015 images of 7,470 lesions retained; no exclusions); Section 2.1 and Table 8 caption for the evaluation cohort (16,133 labelled images split into a 961-

			applicable, a summary of the follow-up time. A diagram may be helpful	image calibration set and a 15,172-image test set). No participant flow diagram is presented because no image was excluded at any stage. Follow-up time does not apply.
	20b	D;E	Report the characteristics overall and, where applicable, for each data source or setting, including the key dates, key predictors (including demographics), treatments received, sample size, number of outcome events, follow-up time, and amount of missing data. A table may be helpful. Report any differences across key demographic groups	Table 1 (development cohort composition by class) and Section 2.1 with the Table 8 caption (evaluation cohort composition). The predictor is the image, so no predictor distribution table applies. Demographic characteristics are not reported: age and sex are present in the HAM10000 metadata but were not used as predictors, and no demographic variable is available for BCN20000 in a form permitting comparison. Differences across demographic groups are therefore not reported, consistent with item 14.
	20c	E	For model evaluation, show a comparison with the development data of the distribution of important predictors (demographics, predictors, and outcome)	Table 8 and its caption, with Table 1. The outcome distribution differs markedly between cohorts and one class (vascular lesions) is absent from the evaluation data; Section 3.8 attributes the collapse in balanced accuracy, alongside preserved discrimination, to this prevalence shift. Predictor distribution is not tabulated because the predictor is the raw image.
**Model development**	21	D;E	Specify the number of participants and outcome events in each analysis (e.g., for model development, hyperparameter tuning, model evaluation)	Section 2.5 and Tables 4, 5 and 8. Per fold: approximately 7,210 training, 800 inner-validation and 2,003 test images, all grouped at the lesion level. Segmentation used 7,010 training, 1,002 validation and 2,003 test images. Explainability used a stratified 300-image subset; the radiomic analysis used 299 of those images, of which 33 are melanomas. External evaluation used 15,172 test images plus a disjoint 961-image calibration split.
**Model specification**	22	D	Provide details of the full prediction model (e.g., formula, code, object, application programming interface) to allow predictions in new individuals and to enable third-party evaluation and implementation, including any restrictions to access or re-use (e.g., freely available, proprietary)	Data Availability Statement, with Sections 2.2, 2.6 and 2.7. Trained weights for all reported configurations, the model-building code and the inference code are freely available under the MIT licence at https://doi.org/10.5281/zenodo.22654523. There are no restrictions on access or re-use of the code or weights. Hardware and software requirements are given in Section 2.2 and in the deposited requirements file.
**Model performance**	23a	D;E	Report model performance estimates with confidence intervals, including for any key subgroups (e.g., sociodemographic). Consider plots to aid presentation	Tables 4, 5, 7 and 8; Figures 5–9. Balanced accuracy is reported with bootstrap BCa 95% confidence intervals; intervals for every metric and fold are in the deposited archive. Calibration is presented as reliability diagrams before and after temperature scaling (Figure 7) and as ECE, MCE and Brier scores. Performance in sociodemographic subgroups is not reported, for the reason given at item 14.
	23b	D;E	If examined, report results of any heterogeneity in model performance across clusters. See TRIPOD-Cluster for additional details	Sections 3.5 and 3.8. Heterogeneity across folds is reported as the input to the Friedman analysis ($N = 5$, $\chi^2 = 7.057$, $p = 0.216$) and is visible in the per-fold confidence intervals of Table 4. Heterogeneity across cohorts is reported in Table 8 as a per-model drop in balanced accuracy of 21.4 to 27.5 points together with a reversal of the internal ranking. No formal cluster-level meta-analytic model was fitted.
**Model updating**	24	E	Report the results from any model updating, including the updated model and subsequent performance	Section 3.8 and Table 8. Re-fitting the temperature on the disjoint BCN20000 calibration split improved calibration for two of six configurations (DenseNet-121 0.0576 → 0.0394; ConvNeXt-T 0.0546 → 0.0459) and worsened it for two others. The re-fitted temperatures are reported and are included in the deposited archive. No refitting of model weights was performed.
**DISCUSSION**				
**Interpretation**	25	D;E	Give an overall interpretation of the main results, including issues of fairness in the context of the objectives and previous studies	Sections 4.1 and 4.2. The four principal findings are stated as negative results and placed against the recent HAM10000 and ISIC 2018 literature, with an explicit statement that the published headline figures are not directly comparable because of differences in splitting protocol and selection. Fairness is addressed in Section 4.4: no claim of equitable performance is made and the absence of phototype data is stated as a limitation.
**Limitations**	26	D;E	Discuss any limitations of the study (such as a non-representative sample, sample size, overfitting, missing data) and their effects on any biases, statistical uncertainty, and generalizability	Section 4.4, which lists: preprocessing components not ablated; parameter count not controlled; limited statistical power at $N = 5$; semi-automatic reference masks; a single external cohort covering six of seven classes with no external segmentation cohort; both cohorts European and specialist-centre with Fitzpatrick III–VI under-represented and no fairness evaluation possible; spectrum and prevalence differences from unselected care; no reader study and therefore no comparison with clinician accuracy; melanoma treated as a homogeneous label despite subtype heterogeneity; residual weakness on the rarest classes; dermoscopy only; retrospective design; metadata not fused.
**Usability of the model in the context of current care**	27a	D	Describe how poor quality or unavailable input data (e.g., predictor values) should be assessed and handled when implementing the prediction model	Partially addressed in Sections 2.3 and 4.5. The preprocessing chain includes hair-artefact removal and colour-constancy correction, which are the two dominant quality problems in dermoscopy, and Figure 3 shows their effect on a real hair-dense image; no image is excluded for quality. Beyond this the manuscript makes no implementation claim: the triage proposal has been withdrawn and no deployment pathway is specified, so no formal input-quality gate or handling rule for unusable images is defined. This is acknowledged as outside the scope of the present evidence.
	27b	D	Specify whether users will be required to interact in the handling of the input data or use of the model, and what level of expertise is required of users	Section 4.5. No autonomous use is proposed. Any use envisaged would require a dermoscopic image acquired by a trained operator and, because threshold-dependent performance transfers poorly, locally labelled data for recalibration at the site of use; the manuscript states explicitly that the model should not be described as plug-

				and-play.
	27c	D;E	Discuss any next steps for future research, with a specific view to applicability and generalizability of the model	Section 4.6. Four next steps follow directly from the negative results: a properly replicated preprocessing ablation across all folds; evaluation of a domain-pretrained dermatology backbone under the present protocol; saliency-localisation metrics reported with matched trivial baselines, and localisation references reflecting dermoscopic criteria rather than lesion extent; and a prospective reader study, without which no claim about the clinical utility of explanations can be made. Section 4.7 adds the regulatory consequences for post-market calibration monitoring.

Note: *Deposited archive: https://doi.org/10.5281/zenodo.22654523.*
